# The proportion of resistant hosts in mixtures should be biased towards the resistance with the lowest breaking cost

**DOI:** 10.1371/journal.pcbi.1011146

**Published:** 2023-05-25

**Authors:** Pauline Clin, Frédéric Grognard, Didier Andrivon, Ludovic Mailleret, Frédéric M. Hamelin

**Affiliations:** 1 Institut Agro, Univ Rennes, INRAE, IGEPP, Rennes, France; 2 Université Côte d’Azur, INRAE, CNRS, ISA, Sophia-Antipolis, France; 3 Université Côte d’Azur, Inria, INRAE, CNRS, Sorbonne Université, Biocore, Sophia-Antipolis, France; University of Zurich, SWITZERLAND

## Abstract

Current agricultural practices facilitate emergence and spread of plant diseases through the wide use of monocultures. Host mixtures are a promising alternative for sustainable plant disease control. Their effectiveness can be partly explained by priming-induced cross-protection among plants. Priming occurs when plants are challenged with non-infective pathogen genotypes, resulting in increased resistance to subsequent infections by infective pathogen genotypes. We developed an epidemiological model to explore how mixing two distinct resistant varieties can reduce disease prevalence. We considered a pathogen population composed of three genotypes infecting either one or both varieties. We found that host mixtures should not contain an equal proportion of resistant plants, but a biased ratio (*e.g.* 80 : 20) to minimize disease prevalence. Counter-intuitively, the optimal ratio of resistant varieties should contain a lower proportion of the costliest resistance for the pathogen to break. This benefit is amplified by priming. This strategy also prevents the invasion of pathogens breaking all resistances.

## Author summary

This study addresses the optimal design of mixtures of resistant hosts to reduce disease prevalence, and prevent the emergence and invasion of multi-virulent pathogen genotypes. Specifically, we investigated how pathogen mediated plant-plant interaction, through immune priming of host defences, influences the optimal proportion of each resistant host in such mixtures. We thus designed a mathematical model explicitly accounting for immune priming in mixtures of two resistant plant varieties. We showed, through analysis and simulation, that the optimal ratio is not 50:50, as commonly done in practice, but should be biased towards the variety that is the least costly for the pathogen to break. We also showed that this bias depends on immune priming effectiveness, and is enhanced when virulence costs are high. This somewhat counter-intuitive outcome finds its explanation in the complex interplay of ecological and physiological mechanisms acting in cultivar mixtures, and is of major relevance for the application of mixtures in agricultural practice. This model therefore provides new clues to best manage and exploit plant biodiversity for sustainable plant health. It also provides new insights into how host heterogeneity and immunity can prevent the evolutionary emergence of pathogens capable of breaking several resistances.

## 1 Introduction

Current agricultural practices can be productive, but they also have negative externalities on our environment. They modify the functioning of ecosystems, contribute to the collapse of biodiversity and to climate change, pollute the environment, and impact human health [1, 2].

Intensive agriculture is mainly based on monocultures [3], which make agricultural environments favorable to plant disease emergence [4]. Despite the continuous selection of resistant plant varieties, pathogens often adapt and quickly break down newly introduced resistances [5]. This is why it is difficult to protect monocultures without pesticides.

One path to developing more sustainable agriculture is through the reintroduction of genetic diversity into crops [6–8]. However, driving diverse host-pathogen populations requires the use of sound ecological concepts and methods from ecology. For instance, the effectiveness of host mixtures against plant diseases involves ecological mechanisms not present in monocultures, such as dilution, interception, competition, and cross-protection effects [9–12]. It has in particular been shown that pathogen competition for susceptible hosts generates apparent cross-protection between host varieties in the mixture [13]. Therefore, an ecologically informed choice of the varieties used in the mixture can allow growers to control plant diseases more effectively.

Immune priming is an additional cross-protection mechanism. Priming occurs when pathogens capable of infecting one variety come in contact with another variety, which they cannot infect. Infection then does not succeed, but the plant increases its level of defense against future infections by the same or other pathogen species [14, 15]. The plant is then said to be primed. Both experimental [16, 17] and theoretical work [13, 18] have highlighted the key effect of priming for the effectiveness of host mixtures against plant diseases, although the ecological mechanisms underlying it have so far only been partially explored.

The density of each variety in the mixture should be carefully chosen [19]. The article [13] considered mixtures of resistant and susceptible hosts exposed to polymorphic pathogen populations (including wild-type and resistance-breaking genotypes), and showed that minimizing disease prevalence is achieved with an intermediate proportion of resistant hosts. This optimal proportion is a direct consequence of immune priming. In a follow-up study, the article [20] considered mixtures with an arbitrary number *n* of resistant varieties, exposed to polymorphic pathogen populations including genotypes capable of breaking several resistances. Due to the large dimension of the model, some simplifying assumptions were made. In particular, all resistant varieties were assumed to be present in the same proportion in the mixture. This assumption is often made in modeling studies [21], and is often translated in practice e.g., [22], though not always e.g., [23]. Our previous study [20] showed that there is a diversity threshold, i.e. a critical number of varieties in the mixture above which the disease can be eradicated in principle. Moreover, priming is expected to improve the efficiency of mixtures by reducing the number of varieties to be mixed in order to remain below a prevalence threshold.

To challenge previous results, we here relaxed the equal proportion assumption, but restricted our analysis to *n* = 2 resistant varieties. We thus considered three pathogen genotypes (two capable of infecting either variety, and one capable of infecting both varieties). Specifically, we wondered (i) whether an imbalanced ratio of varieties in the mixture can minimize disease prevalence, and if so (ii) which of the two varieties should be used in greater proportion.

## 2 Material and methods

Let us consider a mixture of two resistant varieties, each having a single and distinct resistance gene V_*i*_, with *i* = 1, 2. Resistance is qualitative, meaning that an infection either succeeds or fails. Table 1 shows all host-pathogen interactions, following the gene-for-gene model [24]. In this framework, the term “virulence” denotes the pathogen ability to overcome one resistance gene. The pathogen population is composed of at most three pathogen genotypes: two monovirulent genotypes, av_1_/Av_2_ and Av_1_/av_2_, and one doubly virulent genotype, av_1_/av_2_ (Av means “avirulent” and av means “virulent”). Monovirulent pathogen genotypes can infect either V_1_ or V_2_. The doubly virulent pathogen can infect both V_1_ and V_2_. When monovirulent pathogens come in contact with the variety they cannot infect, they trigger priming, which makes the plant partially resistant to future infections by other, compatible pathogens. We ignore the doubly avirulent (Av_1_/Av_2_) pathogen genotype, since it can infect none of the resistant varieties in the mixture, and therefore can not get established in the type of host mixtures considered in this study. The model does not keep track of coinfections for simplicity.

**Table 1:** Host-pathogen interactions in a mixture composed of 2 resistant varieties. Each resistant variety (row) corresponds to a single resistance gene (either V_1_ or V_2_). There are three possible pathogen genotypes that are able to infect at least one variety (columns): av_1_/Av_2_, Av_1_/av_2_ and av_1_/av_2_ (Av means “avirulent” and av means “virulent”). For instance, av_1_/av_2_ means that this pathogen genotype is able to infect both V_1_ and V_2_: this is a doubly virulent pathogen. In contrast, av_1_/Av_2_ and Av_1_/av_2_ cannot infect V_2_ and V_1_, respectively, but instead trigger immune priming on V_2_, and V_1_, respectively. They are monovirulent pathogen genotypes. We ignore the doubly avirulent (Av_1_/Av_2_) pathogen genotype, since it can infect none of the resistant varieties in the mixture, and therefore can not get established in the type of host mixtures considered in this study. The symbol + means infection and the * means priming.

Variety Pathogen	av_1_/Av_2_	Av_1_/av_2_	av_1_/av_2_
V_1_	+	*	+
V_2_	*	+	+

**Fig 1:**
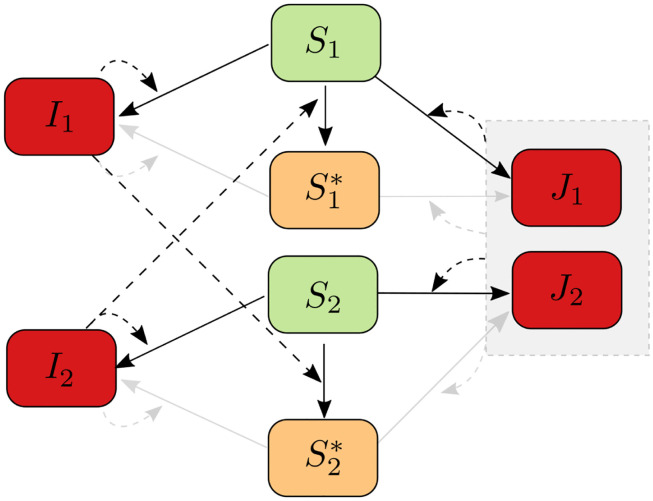
Flow diagram of model 1. *S*_1_ and *S*_2_ are uninfected hosts of varieties V_1_ and V_2_, respectively. $S_{1}^{*}$ and $S_{2}^{*}$ are primed hosts of varieties V_1_ and V_2_, respectively. *I*_1_ and *I*_2_ are hosts of varieties V_1_ and V_2_ (respectively) that are infected by the corresponding monovirulent pathogen genotype. *J*_1_ and *J*_2_ are hosts of varieties V_1_ and V_2_ (respectively) that are infected by the doubly virulent pathogen genotype. The square grouping *J*_1_ and *J*_2_ represents the doubly virulent pathogen genotype. The dashed arrows represent interactions leading to infection or priming. The gray color represents the attenuating effect of priming.

The total host density is *N*, a constant. The proportions of varieties V_1_ and V_2_ are respectively *p*_1_ = 1 − *p* and *p*_2_ = *p*. The density of V_1_ is thus *p*_1_*N* and the density of V_2_ is *p*_2_*N*. The density of uninfected hosts of variety V_*i*_ is *S*_*i*_, for *i* = 1, 2. Similarly, the density of primed hosts of variety V_*i*_ is $S_{i}^{*}$, for *i* = 1, 2. The density of hosts of variety V_*i*_ infected by the corresponding monovirulent pathogen genotype is *I*_*i*_, for *i* = 1, 2. The density of hosts of variety V_*i*_ infected by the doubly virulent pathogen genotype is *J*_*i*_, for *i* = 1, 2. The density of uninfected hosts of variety V_*i*_ is therefore $S_{i}=p_{i}N-S_{i}^{*}-I_{i}-J_{i}$, for *i* = 1, 2. Fig 1 shows a flow diagram of the model.

Bearing a virulence gene (av_*i*_, *i* = 1, 2) involves a cost *c*_*i*_ to the pathogen, reducing its transmission rate by a factor 0 ≤ 1 − *c*_*i*_ ≤ 1, as compared to that, noted *β*, of an avirulent pathogen on a variety with no resistance gene. Monovirulent pathogens therefore have a net transmission rate (1 − *c*_*i*_)*β*, *i* = 1, 2. The idea of a cost as a counterpart of the ability of breaking a resistance gene originated as a theoretical hypothesis to explain the often-observed persistence of virulence polymorphism in pathogen populations, both in agricultural and in wild ecosystems [5, 13, 25–31]. Such a cost has been demonstrated and measured in a number of parasites, including bacteria [32, 33], fungi [34–40], viruses [41–46], nematodes [47] and oomycetes [48].

The virulence (or resistance-breaking) cost is assumed to be multiplicative, meaning that the doubly virulent pathogen bears a fitness cost (1 − *c*_1_)(1 − *c*_2_) as compared to an avirulent genotype on a variety with no resistance gene [20, 26, 49–55]. Doubly virulent pathogens therefore have a net transmission rate (1 − *c*_1_)(1 − *c*_2_)*β* from both varieties in the mixture.

The priming effect, 0 ≤ *ρ* ≤ 1, reduces the probability that a primed host is infected by a virulent pathogen genotype compared to a non-primed host. Experimental evidence suggests that priming usually becomes effective a few hours or days after challenge with an avirulent pathogen genotype [56, 57]. However, we here ignore this delay for simplicity. In some cases, priming can be fully effective (i.e., *ρ* = 1) [58]. Then, a virulent pathogen genotype cannot infect a primed host as long as priming is active.

The rate at which priming loses its efficiency is *γ*. It corresponds to the inverse of the mean time during which priming is effective. Several studies have shown that priming can last for several weeks. The original one [56], on the tobacco mosaic virus, estimates that it persists for 20 days, but broader studies including viruses, bacteria, and fungi show that it can last for weeks to months [58, 59]. Priming has been described in many plant species and is likely to be ubiquitous in higher plants. The main biological models for study of priming include tobacco, cucumber, and Arabidopsis [60].

For simplicity, we consider a continuous-time model with continuous planting and replanting best adapted to perennial crops in tropical regions [61]. More specifically, we consider that the host is present yearlong, and we ignore seasonality in climatic conditions.

Infected hosts remain infectious until harvest, as is the case for most plant viruses and many other parasites. The rate at which a host is replaced with an uninfected one (due to harvesting and replanting) is *α*. It corresponds to the inverse of the length of the growing period.

The model is then expressed as a system of six ordinary differential equations:

$$\begin{array}{ccc} {\dot{I}}_{1} & = & \left(1-c_{1}\right)\beta I_{1}S_{1}+\left(1-\rho \right)\left(1-c_{1}\right)\beta I_{1}S_{1}^{*}-\alpha I_{1}\;,\\ {\dot{I}}_{2} & = & \left(1-c_{2}\right)\beta I_{2}S_{2}+\left(1-\rho \right)\left(1-c_{2}\right)\beta I_{2}S_{2}^{*}-\alpha I_{2}\;,\\ {\dot{J}}_{1} & = & \left(1-c_{1}\right)\left(1-c_{2}\right)\beta \left(J_{1}+J_{2}\right)S_{1}+\left(1-\rho \right)\left(1-c_{1}\right)\left(1-c_{2}\right)\beta \left(J_{1}+J_{2}\right)S_{1}^{*}-\alpha J_{1}\;,\\ {\dot{J}}_{2} & = & \left(1-c_{1}\right)\left(1-c_{2}\right)\beta \left(J_{1}+J_{2}\right)S_{2}+\left(1-\rho \right)\left(1-c_{1}\right)\left(1-c_{2}\right)\beta \left(J_{1}+J_{2}\right)S_{2}^{*}-\alpha J_{2}\;,\\ {\dot{S}}_{1}^{*} & = & \left(1-c_{2}\right)\beta I_{2}S_{1}-\left(1-\rho \right)\left(1-c_{1}\right)\beta I_{1}S_{1}^{*}-\left(1-\rho \right)\left(1-c_{1}\right)\left(1-c_{2}\right)\beta \left(J_{1}+J_{2}\right)S_{1}^{*}-\left(\gamma +\alpha \right)S_{1}^{*}\;,\\ {\dot{S}}_{2}^{*} & = & \left(1-c_{1}\right)\beta I_{1}S_{2}-\left(1-\rho \right)\left(1-c_{2}\right)\beta I_{2}S_{2}^{*}-\left(1-\rho \right)\left(1-c_{1}\right)\left(1-c_{2}\right)\beta \left(J_{1}+J_{2}\right)S_{2}^{*}-\left(\gamma +\alpha \right)S_{2}^{*}\;, \end{array}$$ (1)

where the dot denotes differentiation with respect to time *t*.

To simplify the analysis and reduce the number of parameters, we rescale variables and parameters in this way:

$$\begin{array}{c} y_{1}=\frac{I_{1}}{N}\;,\;y_{2}=\frac{I_{2}}{N}\;,\;z_{1}=\frac{J_{1}}{N}\;,\;z_{2}=\frac{J_{2}}{N}\;,\;m_{1}=\frac{S_{1}^{*}}{N}\;,\;m_{2}=\frac{S_{2}^{*}}{N}\;, \end{array}$$

and

$$\begin{array}{c} t^{*}=\alpha t\;,\;R=\frac{\beta N}{\alpha }\;,\;\nu =\frac{\gamma +\alpha }{\alpha }\geq 1\;. \end{array}$$

More specifically, *R* is the basic reproductive number of an avirulent pathogen on a variety with no resistance gene. We therefore assume *R* > 1, otherwise the disease would not persist (if an avirulent pathogen cannot persist in a pure susceptible stand, a virulent pathogen cannot persist either, due to virulence costs). The re-scaled removal rate *ν* means that primed hosts can be removed due to either harvest or loss of priming.

A dimensionless version of model (1) is the following,

$$\begin{array}{ccc} y_{1}^{\prime } & = & \left(1-c_{1}\right)Ry_{1}\left(1-p-m_{1}-y_{1}-z_{1}\right)+\left(1-\rho \right)\left(1-c_{1}\right)Ry_{1}m_{1}-y_{1}\;,\\ y_{2}^{\prime } & = & \left(1-c_{2}\right)Ry_{2}\left(p-m_{2}-y_{2}-z_{2}\right)+\left(1-\rho \right)\left(1-c_{2}\right)Ry_{2}m_{2}-y_{2}\;,\\ z_{1}^{\prime } & = & \left(1-c_{1}\right)\left(1-c_{2}\right)R\left(z_{1}+z_{2}\right)\left(1-p-m_{1}-y_{1}-z_{1}\right)+\left(1-\rho \right)\\ & & \left(1-c_{1}\right)\left(1-c_{2}\right)R\left(z_{1}+z_{2}\right)m_{1}-z_{1}\;,\\ z_{2}^{\prime } & = & \left(1-c_{1}\right)\left(1-c_{2}\right)R\left(z_{1}+z_{2}\right)\left(p-m_{2}-y_{2}-z_{2}\right)+\left(1-\rho \right)\\ & & \left(1-c_{1}\right)\left(1-c_{2}\right)R\left(z_{1}+z_{2}\right)m_{2}-z_{2}\;,\\ m_{1}^{\prime } & = & \left(1-c_{2}\right)Ry_{2}\left(1-p-m_{1}-y_{1}-z_{1}\right)-\left(1-\rho \right)\left(1-c_{1}\right)Ry_{1}m_{1}-\left(1-\rho \right)\\ & & \left(1-c_{1}\right)\left(1-c_{2}\right)R\left(z_{1}+z_{2}\right)m_{1}-\nu m_{1}\;,\\ m_{2}^{\prime } & = & \left(1-c_{1}\right)Ry_{1}\left(p-m_{2}-y_{2}-z_{2}\right)-\left(1-\rho \right)\left(1-c_{2}\right)Ry_{2}m_{2}-\left(1-\rho \right)\\ & & \left(1-c_{1}\right)\left(1-c_{2}\right)R\left(z_{1}+z_{2}\right)m_{2}-\nu m_{2}\;, \end{array}$$ (2)

where the prime denotes differentiation with respect to time t*.

The prevalence of the disease is defined as the proportion of infectious hosts in the mixture:

$$\begin{array}{c} P=\frac{I_{1}+I_{2}+J_{1}+J_{2}}{N}=y_{1}+y_{2}+z_{1}+z_{2}\;. \end{array}$$

A necessary condition for a monovirulent pathogen to invade an uninfected susceptible population (and for that matter a mixture) is that its basic reproductive number in a pure susceptible stand, *R*_*i*_ = *R*(1 − *c*_*i*_), *i* = 1, 2, exceeds 1. Similarly, *R*_3_ = *R*(1 − *c*_1_)(1 − *c*_2_) is the basic reproductive number of the doubly virulent pathogen. Model parameters and variables are listed in Table 2.

**Table 2:** Model parameters and variables.

Parameter	Definition
V_*i*_	variety with a single resistance gene, *i* = 1, 2
*p*_*i*_	proportion of each variety in the mixture: *p*_*i*_ ∈ [0, 1] for *i* = 1, 2
*p*	proportion of variety 2 in the mixture, i.e. *p* = *p*_2_ = 1 − *p*1
*c*_*i*_	virulence cost for each virulence: *c*_*i*_ ∈ [0, 1] for *i* = 1, 2
*ρ*	priming effect: *ρ* ∈ [0, 1]
*γ*	priming loss rate: *γ* ≥ 0
*α*	harvest and replanting rate: *α* > 0
*β*	pathogen transmission rate: *β* > 0
*N*	total host population density: *N* > 0
*R*	basic reproductive number of an avirulent pathogen in a pure susceptible stand: *R* = *βN*/*α* > 1
*R*_*i*_	basic reproductive number of a monovirulent pathogen in a pure susceptible stand: *R*_*i*_ = *R*(1 − *c*_*i*_), for *i* = 1, 2
*R*_3_	basic reproductive number of the doubly virulent pathogen: *R*_3_ = *R*(1 − *c*_1_)(1 − *c*_2_)
*ν*	re-scaled removal rate: *ν* = (*γ* + *α*)/*α* ≥ 1
Variable	Definition
*t*	time: *t* ≥ 0
*I*_*i*_	density of hosts of variety *i* infected by the associated monovirulent pathogen genotype, *i* = 1, 2
*J*_*i*_	density of hosts of variety *i* infected by the doubly virulent pathogen genotype, *i* = 1, 2
$S_{i}^{*}$	density of hosts of variety *i* that are primed, *i* = 1, 2
*S*_*i*_	density of hosts of variety *i* that are uninfected, *i* = 1, 2
*y*_*i*_	proportion of hosts of variety *i* infected by the associated monovirulent pathogen genotype: *y*_*i*_ = *I*_*i*_/*N*
*z*_*i*_	proportion of hosts of variety *i* infected by the doubly virulent pathogen genotype: *z*_*i*_ = *J*_*i*_/*N*
*m*_*i*_	proportion of hosts of variety *i* that are primed: $m=S_{i}^{*}/N$

The model was only partly amenable to mathematical analysis (see S1 Text). There are 7 biologically feasible equilibria:

1. the “disease-free” equilibrium (0, 0, 0, 0, 0, 0),
2. the “monovirulent 1” equilibrium (*y*_1_, 0, 0, 0, 0, *m*_2_),
3. the “monovirulent 2” equilibrium (0, *y*_2_, 0, 0, *m*_1_, 0),
4. the “doubly virulent” equilibrium (0, 0, *z*_1_, *z*_2_, 0, 0),
5. the “monovirulent 1 and doubly virulent” equilibrium (*y*_1_, 0, *z*_1_, *z*_2_, 0, *m*_2_),
6. the “monovirulent 2 and doubly virulent” equilibrium (0, *y*_2_, *z*_1_, *z*_2_, *m*_1_, 0),
7. the “monovirulent 1 and monovirulent 2” equilibrium (*y*_1_, *y*_2_, 0, 0, *m*_1_, *m*_2_).

In particular, there exists no equilibrium of the form (*y*_1_, *y*_2_, *z*_1_, *z*_2_, *m*_1_, *m*_2_) > 0, in which the three possible pathogen genotypes coexist (section S1.1 in S1 Text).

All the positiveness conditions of the equilibria were obtained analytically, except for the “monovirulent 1 and monovirulent 2” equilibrium, for which only sufficient conditions were obtained (Table 3; section S1 in S1 Text). Regarding the stability conditions, explicit expressions were obtained for the disease-free, monovirulent 1, monovirulent 2, and doubly virulent equilibria. However, we were not able to derive explicit stability conditions for the “monovirulent *i* and doubly virulent” equilibria (*i* = 1, 2), and the “monovirulent 1 and monovirulent 2” equilibrium.

**Table 3:** Summary of model (2) equilibria, with their positiveness and stability conditions. All conditions are necessary and sufficient conditions except those marked with an exclamation mark, which are only sufficient conditions in general. The expressions of ${\hat{\rho }}_{1},{\hat{\rho }}_{2},{\overset{ˇ}{\rho }}_{1},{\overset{ˇ}{\rho }}_{2}$ are given by equations S5, S7, S13, S14 in S1 Text, respectively. The exclamation mark means that we have no explicit conditions in general. The meaning of the parameters can be found in Table 2. The “extra” stability conditions are those that are not redundant with the positiveness conditions.

#	Equilibrium	Positiveness conditions	Extra Stability conditions
1	(0, 0, 0, 0, 0, 0)	None	*R*_1_(1 − *p*), *R*_2_*p*, *R*_3_ < 1
2	(*y*_1_, 0, 0, 0, 0, *m*_2_)	*R*_1_(1 − *p*) > 1	$\rho >{\hat{\rho }}_{1}\;,\;{\overset{ˇ}{\rho }}_{1}$
3	(0, *y*_2_, 0, 0, *m*_1_, 0)	*R*_2_*p* > 1	$\rho >{\hat{\rho }}_{2}\;,\;{\overset{ˇ}{\rho }}_{2}$
4	(0, 0, *z*_1_, *z*_2_, 0, 0)	*R*_3_ > 1	*R*_3_ > *R*_1_(1 − *p*), *R*_2_*p*
5	(*y*_1_, 0, *z*_1_, *z*_2_, 0, *m*_2_)	*R*_1_(1 − *p*) > *R*_3_ > 1 and $\rho <{\hat{\rho }}_{1}$	?
6	(0, *y*_2_, *z*_1_, *z*_2_, *m*_1_, 0)	*R*_2_*p* > *R*_3_ > 1 and $\rho <{\hat{\rho }}_{2}$	?
7	(*y*_1_, *y*_2_, 0, 0, *m*_1_, *m*_2_)	*R*_1_(1 − *p*), *R*_2_*p* > 1 (!)	?

We thus complemented the analysis with numerical computations. By using the expressions of the different equilibria and the Jacobian matrix of the model, we numerically assessed the stability of the equilibria for chosen parameter sets. More specifically, parameter sets were chosen by varying one parameter at a time around an arbitrary default parameter set: *R* = 7, *ν* = 1, *ρ* = 0.8, *c*_1_ = *c*_2_ = 0.4. The parameter values considered were *R* = {2, 3, 4, 5, 6, 7, 10}, *ν* = {1, 2, 10}, *ρ* = {0, .2, .5, .7, .8, .9, .95, 1}, *c*_1_, *c*_2_ = {.1, .2, .3, .35, .4, .45, .5, .55, .6, .65, .7, .75}.

This way, we obtained a rather extensive picture of the model behavior. This approach allowed us to plot the prevalence of the disease (*P*) at equilibrium as a function of the proportion (*p*) of the second variety (resistance 2), while keeping track of the genotypic composition of the pathogen population. In what follows, we restrict the results to the biologically reasonable case in which *c*_1_, *c*_2_ ≤ 0.5, meaning that the virulence cost is reasonably low.

## 3 Results

The model yields four possible outcomes: disease extinction, the persistence of a single monovirulent pathogen genotype (either Av_1_/av_2_ or av_1_/Av_2_), the persistence of the doubly virulent pathogen (av_1_/av_2_) while monovirulent pathogen genotypes are excluded, and the coexistence of a monovirulent pathogen with the doubly virulent pathogen genotype (either Av_1_/av_2_ and av_1_/av_2_, or av_1_/Av_2_ and av_1_/av_2_).

The coexistence of the three possible genotypes is impossible (section S1.1 in S1 Text). This may be interpreted as an instance of the competitive exclusion principle, which states that three species (here pathogen genotypes) cannot coexist on fewer than 3 resources (2 host varieties here) [62].

Moreover, the coexistence of the two monovirulent pathogens is likely impossible (assuming *c*_1_, *c*_2_ ≤ 0.5; equation S16 in S1 Text) since the virulence costs allow the doubly virulent pathogen genotype to persist, and the latter likely excludes the monovirulent pathogen genotype capable of infecting the variety that is in the lowest proportion. This is because there are no coinfections in the model, therefore hosts infected by the doubly virulent pathogen are no longer available for the monovirulent pathogens.

Table 3 summarizes the equilibria, their existence/positiveness and stability conditions. In a mixture of two resistant varieties, the quantities (1 − *p*)*R*_1_, *pR*_2_, and *R*_3_ are the basic reproductive numbers of the monovirulent 1 (which can infect a fraction 1 − *p* of the host population), the monovirulent 2 (which can infect a fraction *p* of the host population), and the doubly virulent (which can infect the entire host population), respectively. If the basic reproductive number of a pathogen genotype is lower than one, the latter cannot persist in the mixture. The doubly virulent equilibrium is stable if and only if the basic reproductive number of the doubly virulent pathogen is greater than the basic reproductive numbers of the monovirulents pathogens (in the mixture). A necessary condition for the “monovirulent *i* and doubly virulent” equilibria (*i* = 1, 2) to be positive is that the basic reproductive number of the monovirulent pathogen *i* is greater than that of the doubly virulent pathogen.

### Even if varieties are epidemiologically interchangeable (*c*_1_ = *c*_2_), they should not be mixed in equal proportions to minimize disease prevalence

**Fig 2:**
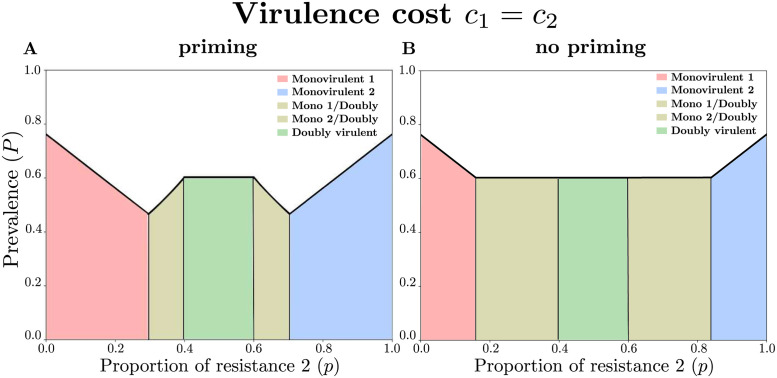
Prevalence of the disease (*P*, black line) at equilibrium as a function of the proportion of resistance 2 (*p*), when varieties epidemiologically interchangeable (*c*_1_ = *c*_2_). (**A**) When priming occurs (*ρ* = 0.7), the optimal proportion deviates from *p* = 0.5. (**B**) In absence of priming (*ρ* = 0), the disease prevalence is minimized for a range of *p* values. The colored areas correspond to different genetic compositions of the pathogen population at equilibrium. From left to right: monovirulent 1 only, coexistence of monovirulent 1 and doubly virulent, doubly virulent only, coexistence of monovirulent 2 and doubly virulent, and monovirulent 2 only. Parameter values: *R* = 7, *ν* = 1, and *c*_1_ = *c*_2_ = 0.4. The prevalences are the same at the edges (*p* = 0 and *p* = 1, in which one or the other monovirulent genotype is present) or in the middle region (in which only the doubly virulent genotype is present) regardless of whether priming occurs (**A**) or not (**B**). Priming only has an effect in the intermediate regions, in which a monovirulent genotype coexists with the doubly virulent one (in the absence of priming).

**Fig 3:**
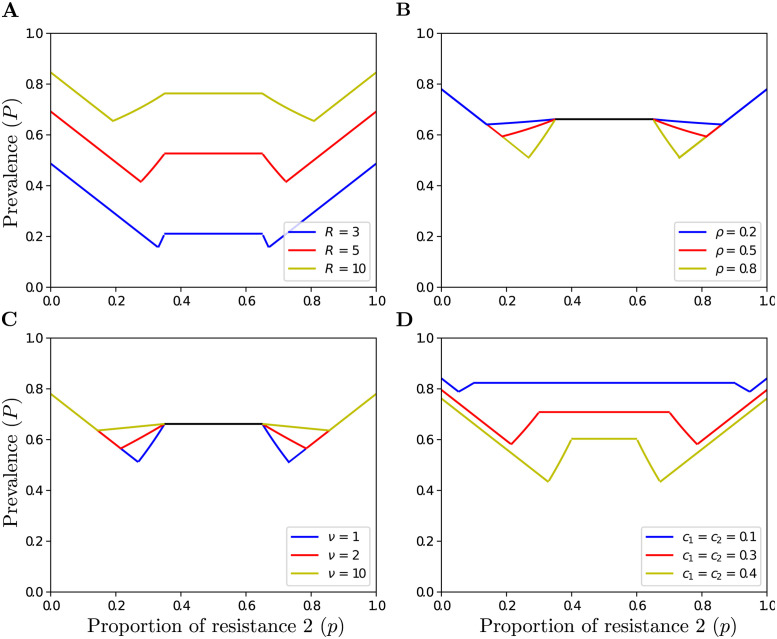
Prevalence of the disease (*P*) at equilibrium as a function of the proportion of resistance 2 (*p*), when varieties are epidemiologically interchangeable (*c*_1_ = *c*_2_ < 0.5) for different values of (**A**) the basic reproductive number *R*, (**B**) the priming effect *ρ*, (**C**) the removal rate *ν*, (**D**) and the virulence costs *c*_1_, and *c*_2_. Parameter values: (**A**) *ρ* = 0.7, *ν* = 1, and *c*_1_ = *c*_2_ = 0.35, (**B**) *R* = 7, *ν* = 1, and *c*_1_ = *c*_2_ = 0.35, (**C**) *R* = 7, *ρ* = 0.8, and *c*_1_ = *c*_2_ = 0.35, (**D**) and *R* = 7, *ρ* = 0.8, and *ν* = 1.

Fig 2A shows that a balanced ratio (50:50) of resistant varieties (*p* = 0.5) does not minimize the prevalence of the disease. Instead, the minimum of prevalence is reached for 70:30 and 30:70 (variety 1:variety 2) ratios, for this specific parameter set. These ratios correspond to thresholds at which the doubly virulent pathogen can invade. The optimal strategy is therefore to mix the varieties as much as possible while preventing the doubly virulent pathogen to invade. Fig 2B shows that this result is a direct consequence of priming: in the absence of priming, any ratio between 82:18 and 18:82 minimizes disease prevalence, for this parameter set. However, the most extreme optimal ratios (about 82:18 and 18:82) prevent the emergence of the doubly virulent pathogen, in the absence of priming. Varieties should thus not be mixed in equal proportions, whether or not priming occurs. The results illustrated in Fig 2 are representative of the results obtained with a broader range of parameter values, as shown in Fig 3.

### If varieties are not epidemiologically interchangeable (*c*_1_ ≠ *c*_2_), the mixture should be biased against the resistance that has the highest breaking cost

**Fig 4:**
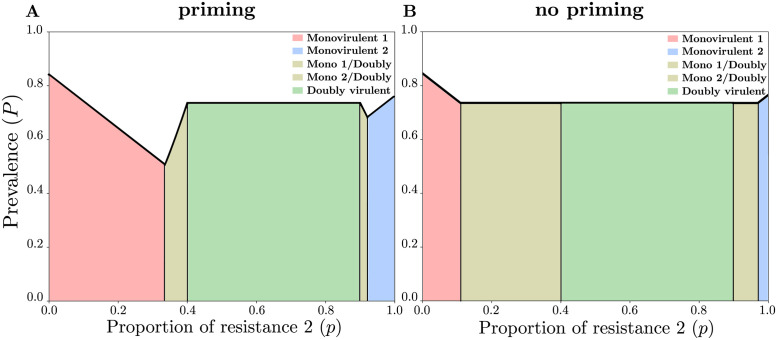
Prevalence of the disease (*P*, black line) at equilibrium as a function of the proportion of resistance 2 (*p*), when varieties are not epidemiologically interchangeable (*c*_1_ ≠ *c*_2_). (**A**) When priming occurs (*ρ* = 0.9), there is a unique optimal proportion biased towards the variety the most likely to be broken, here variety 1 (assumming *c*_1_ < *c*_2_). (**B**) In absence of priming (*ρ* = 0), the disease prevalence is minimized for a range of *p* values. The colored areas correspond to different genetic compositions of the pathogen population at equilibrium. From left to right: monovirulent 1 only, coexistence of monovirulent 1 and doubly virulent, doubly virulent only, coexistence of monovirulent 2 and doubly virulent, and monovirulent 2 only. Parameter values: *R* = 7, *ν* = 1, *c*_1_ = 0.1, and *c*_2_ = 0.4.

**Fig 5:**
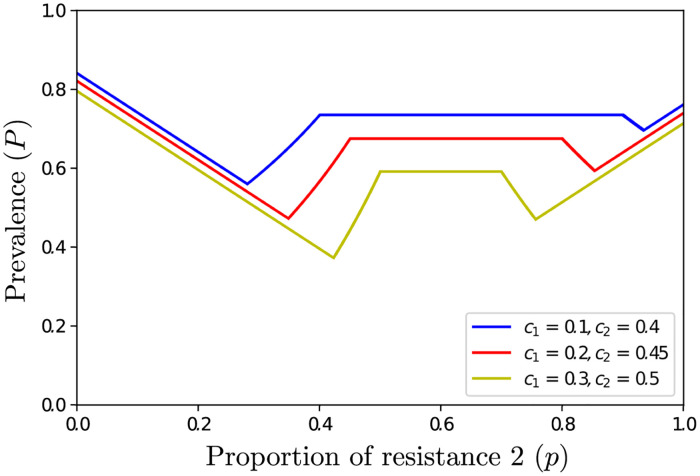
Prevalence of the disease (*P*) at equilibrium as a function of the proportion of resistance 2 (*p*), when varieties are not epidemiologically interchangeable (*c*_1_ ≠ *c*_2_ < 0.5). Parameter values: *R* = 7, *ρ* = 0.8, and *ν* = 1.

Fig 4A shows that a unique ratio (65:35, for the considered parameter set), of (variety 1:variety 2) resistant plants minimizes disease prevalence, when *c*_1_ < *c*_2_. The optimal strategy, that consists in mixing varieties as much as possible while preventing the doubly virulent to invade, is biased against resistant variety 2, which has the greatest breaking cost (since *c*_1_ < *c*_2_). This contrasts with the optimal variety to be used in monoculture, which is variety 2 (compare the prevalences for *p* = 0 and *p* = 1). Fig 4B shows that, even in absence of priming, mixing varieties as much as possible while preventing the doubly virulent pathogen to invade is achieved by using variety 1 in greater proportion than variety 2 (compare the sizes of the “monovirulent 1” and “monovirulent 2” areas). The results illustrated in Fig 4 are representative of the results obtained with a broader range of parameter values, as show in Fig 5 for other virulence costs.

We developed an interactive interface allowing the user to test their own parameter sets: https://paulineclin-2-resistants-priming-model-app-chap3-wuqamb.streamlit.app/.

## 4 Discussion

The fight against plant diseases requires sustainable, environmentally friendly solutions. Host mixtures are part of the ecological solution [63]. They have been the object of a vast literature corpus for decades [9], including a number of modeling studies [64]. However, the basic mathematical theory underlying the effectiveness of host mixtures against plant diseases is still little developed [13, 19, 20]. In this study, we theoretically explored, for the first time, the effectiveness of host mixtures composed of two qualitatively resistant varieties in variable proportions, against monovirulent and doubly virulent pathogen genotypes.

We first explored whether resistant varieties that differ only in their single specific resistance gene, and that are otherwise epidemiologically interchangeable, should be mixed in equal proportions. Somewhat unexpectedly, we found that the equi-proportion mixtures were not the optimal ones. Fig 2A shows a case where the optimal ratio is around 30:70. While a 50:50 ratio reduces the disease prevalence compared to a monoculture, it is not optimal. This is because it selects for a doubly virulent pathogen, that can infect both resistances in the mixture. However, it is possible to mix varieties in such a way that the doubly virulent pathogen does not invade. More specifically, with the parameters of Fig 2A, the 30:70 ratio is the threshold above which the doubly virulent pathogen can invade. The optimal strategy is thus to mix varieties as much as possible but still so that the doubly virulent pathogen does not invade. This way, one of the two varieties cannot be infected at all by the pathogen population (composed of an incompatible, monovirulent pathogen), which explains that the 30:70 ratio performs better than monocultures (selecting for the corresponding monovirulent pathogen) or the 50:50 ratio (selecting for the doubly virulent pathogen). However, this result is a direct consequence of immune priming. If priming does not occur, then any ratio from 18:82 to 50:50 minimizes disease prevalence (Fig 2B), for this parameter set. Nevertheless, one may argue that the most biased optimal ratio (18:82 for this parameter set) is more durable, since it prevents the emergence of the doubly virulent pathogen, and therefore maintains one of the resistances effective in the long run. When there is priming, an optimum that maximizes cross-protection between varieties emerges. That is, the variety infected by the corresponding monovirulent pathogen indirectly protects the other variety through priming. The latter variety, which is not infected, protects the former through a dilution effect [65].

Next, given that varieties should not be mixed in equal proportions, we wondered which variety should be used in greater proportion in the mixture. In this study, we focused on qualitative resistances only, meaning that the varieties do not differ in terms of quantitative resistance. Therefore, the only possible epidemiological difference between varieties is the virulence (or resistance-breaking) cost associated with the single, specific resistance gene each carries. This way, we assumed that one variety is more costly for the pathogen to infect. One might think that, to minimize disease prevalence, the latter variety should be mixed in greater proportion than the variety which is less costly for the pathogen to infect. Surprisingly, we found the reverse result: the variety that is less costly for the pathogen to infect should be mixed in greater proportion than the other variety (Fig 4A). This is because the optimal strategy is to mix the varieties as much as possible while preventing the doubly virulent pathogen to invade, and it is easier to prevent the invasion of the most costly virulence (Fig 4B shows that the “monovirulent 1” area is greater than the “monovirulent 2” area, with *c*_2_ > *c*_1_). For the more costly virulence not to be selected for, one should mix the associated resistance in lower proportion than the resistance the least costly to break. In addition, the monovirulent pathogen bearing the least costly virulence is more transmissible, which therefore increases priming-induced cross-protection. This further prevents the doubly virulent pathogen from invading. More specifically, if the costliest variety is rare, the most transmissible pathogen (capable of infecting the least costly variety) is selected for, which produces more spill-over and therefore priming on the costliest variety. This in turns allows for a larger proportion of the costliest variety without emergence of the double virulence. By contrast, if the least costly variety is rare, the least transmissible pathogen is selected for, which leads to lower spill-over and therefore less priming.

We have been unable to find actual experimental data allowing us to challenge the outputs of our model. Indeed, we have found no reports of experiments involving varying proportions of resistant hosts in two-way mixtures with no susceptible component. Most reports on two way mixtures relate to combination of a susceptible and one resistant host [12, 66]: these preclude the intervention of some of the mechanisms (such as the competition between monovirulent and doubly virulent genotypes) that are at the core of our model.

Choosing the optimal proportion of resistant components in host mixtures is critical for the lasting performance of this disease control strategy. One key output of our theoretical work is that the optimal proportion for reducing disease prevalence is also the optimal proportion for protecting the resistance genes involved in the mixture, by preventing the spread and persistence of doubly virulent pathogen genotypes. This conclusion, based on the graphical analysis shown in Figs 2–5, is of great practical relevance for the design of mixtures in perennial hosts, in which altering the proportion of mixture components is not possible during the commercial life of the stand [67]. Therefore, we expect that our theoretical results and the online interface that we designed will be of great value in perennial crops, where mixture composition is set once and for all [68].

In this study, we focused on relatively low virulence (or resistance-breaking) costs (*c*_1_, *c*_2_ ≤ 0.5), as is generally the case [69]. However, these costs might be higher in some virus and nematode species [43, 47]. In this case (*c*_1_, *c*_2_ > 0.5), a balanced (50:50) ratio of resistant varieties can be within the optimal range, assuming the varieties are epidemiologically interchangeable (*c*_1_ = *c*_2_, Fig C in S1 Text). This is because the virulent costs are so high that the doubly virulent pathogen genotype cannot persist. Instead, the monovirulent genotypes may coexist. However, even in this case, a biased ratio may perform equally well as a balanced 50:50 ratio. Nevertheless, if the varieties are not epidemiologically interchangeable while both virulence costs are relatively high (*c*_2_ > *c*_1_ > 0.5), the optimal ratio can be slightly biased against the resistance with the lowest breaking cost (Fig D in S1 Text). These results contrast with those obtained for reasonably low virulence costs (*c*_1_ ≤ *c*_2_ ≤ 0.5; Figs 1 and 2). However, relatively low virulence costs represent the most generic situation in the state of our knowledge to date.

To go further in the study of host mixtures, it would be relevant to consider a third variety in the mixture, to explore whether the bias would decrease as the number of varieties increases. This would be a way of better connecting this study to a previous one [20], in which we considered an arbitrary number of varieties in equal proportions. It would also be interesting to explore whether our main result (the optimal mixture is biased towards the resistance with the lowest breaking cost) extends to other traits than the breaking cost, for instance the probability that one of the resistances is broken down. This is left for future research.

To conclude, our model shows that, to minimize the prevalence of the disease, mixing resistant varieties in equal proportions is sub-optimal, and that the optimal proportions directly depend on the virulence costs associated with each resistance. This study therefore shows the importance of assessing the virulence costs associated with each resistance when designing a successful and lasting mixture. It also offers a number of practical indications on designing such simple mixtures for maximum performance, and best ecological sustainability, especially in perennial crops for which optimal design is critical.

## Supporting information

S1 Text Mathematical and numerical appendices. (PDF)

## References

[1] Tilman D., Cassman K. G., Matson P. A., Naylor R., and Polasky S. (2002). Agricultural sustainability and intensive production practices. *Nature*, 418(6898):671–677. doi: 10.1038/nature01014 12167873

[2] Campbell B. M., Beare D. J., Bennett E. M., Hall-Spencer J. M., Ingram J. S., Jaramillo F., and al (2017). Agriculture production as a major driver of the earth system exceeding planetary boundaries. *Ecol. Soc*, 22(4).

[3] Wolfe M. S. and Ceccarelli S. (2020). The increased use of diversity in cereal cropping requires more descriptive precision. *J. Sci. Food Agric*, 100(11):4119–4123. doi: 10.1002/jsfa.9906 31271220

[4] Fones H. N., Bebber D. P., Chaloner T. M., Kay W. T., Steinberg G., and Gurr S. J. (2020). Threats to global food security from emerging fungal and oomycete crop pathogens. *Nat. Food*, 1(6):332–342. doi: 10.1038/s43016-020-0075-0 37128085

[5] Brown J. K. (2015). Durable resistance of crops to disease: a darwinian perspective. *Annu. Rev. Phytopathol*, 53:513–539.. doi: 10.1146/annurev-phyto-102313-045914 26077539

[6] Barot S., Allard V., Cantarel A., Enjalbert J., Gauffreteau A., Goldringer I., and al (2017). Designing mixtures of varieties for multifunctional agriculture with the help of ecology. a review. *Agron. Sustain. Dev*, 37(2):1–20. doi: 10.1007/s13593-017-0418-x

[7] Yang L.-N., Pan Z.-C., Zhu W., Wu E., He D.-C., Yuan X., Qin Y.-Y., Wang Y., Chen R.-S., Thrall P. H., et al. (2019). Enhanced agricultural sustainability through within-species diversification. *Nature Sustainability*, 2(1):46–52. doi: 10.1038/s41893-018-0201-2

[8] Montazeaud G., Rousset F., Fort F., Violle C., Fréville H., and Gandon S. (2020). Farming plant cooperation in crops. *Proc. Royal Soc. B*, 287(1919):20191290. doi: 10.1098/rspb.2019.1290 31964305 PMC7015324

[9] Mundt C. C. (2002). Use of multiline cultivars and cultivar mixtures for disease management. *Annu. Rev. Phytopathol*, 40(1):381–410. doi: 10.1146/annurev.phyto.40.011402.113723 12147765

[10] Garrett K. A., Zúñiga L., Roncal E., Forbes G., Mundt C., Su Z., and al (2009). Intraspecific functional diversity in hosts and its effect on disease risk across a climatic gradient. *Ecol. Appl*, 19(7):1868–1883. doi: 10.1890/08-0942.1 19831076

[11] Reiss E. R. and Drinkwater L. E. (2018). Cultivar mixtures: a meta-analysis of the effect of intraspecific diversity on crop yield. *Ecol. Appl*, 28(1):62–77. doi: 10.1002/eap.1629 28940830

[12] Orellana-Torrejon C., Vidal T., Boixel A.-L., Gélisse S., Saint-Jean S., and Suffert F. (2022). Annual dynamics of zymoseptoria tritici populations in wheat cultivar mixtures: A compromise between the efficacy and durability of a recently broken-down resistance gene? *Plant Pathol*, 71(2):289–303. doi: 10.1111/ppa.13458

[13] Clin P., Grognard F., Mailleret L., Val F., Andrivon D., and Hamelin F. M. (2021). Taking advantage of pathogen diversity and immune priming to minimize disease prevalence in host mixtures: a model. *Phytopathology*, 111(7):1219–1227. doi: 10.1094/PHYTO-09-20-0429-R 33297731

[14] Finckh M., Gacek E., Goyeau H., Lannou C., Merz U., Mundt C., and al (2000). Cereal variety and species mixtures in practice, with emphasis on disease resistance. *Agronomie*, 20(7):813–837. doi: 10.1051/agro:2000177

[15] Gourbal B., Pinaud S., Beckers G. J., Van Der Meer J. W., Conrath U., and Netea M. G. (2018). Innate immune memory: An evolutionary perspective. *Immunol. Rev*, 283(1):21–40. doi: 10.1111/imr.12647 29664574

[16] Calonnec A., Goyeau H., and de Vallavieille-Pope C. (1996). Effects of induced resistance on infection efficiency and sporulation of puccinia striiformis on seedlings in varietal mixtures and on field epidemics in pure stands. *Eur. J. Plant Pathol*, 102(8):733–741. doi: 10.1007/BF01877147

[17] Lannou C., Hubert P., and Gimeno C. (2005). Competition and interactions among stripe rust pathotypes in wheat-cultivar mixtures. *Plant Pathol*, 54(5):699–712. doi: 10.1111/j.1365-3059.2005.01251.x

[18] Lannou C., De Vallavieille-Pope C., and Goyeau H. (1995). Induced resistance in host mixtures and its effect on disease control in computer-simulated epidemics. *Plant Path*, 44(3):478–489. doi: 10.1111/j.1365-3059.1995.tb01670.x

[19] Mikaberidze A., McDonald B., and Bonhoeffer S. (2015). Developing smarter host mixtures to control plant disease. *Plant Pathol*, 64(4):996–1004. doi: 10.1111/ppa.12321

[20] Clin P., Grognard F., Andrivon D., Mailleret L., and Hamelin F. M. (2022). Host mixtures for plant disease control: Benefits from pathogen selection and immune priming. *Evol. Appl*. doi: 10.1111/eva.13386 35782013 PMC9234633

[21] Chabas H., Lion S., Nicot A., Meaden S., van Houte S., Moineau S., and al (2018). Evolutionary emergence of infectious diseases in heterogeneous host populations. *PLoS Biol*, 16(9):e2006738. doi: 10.1371/journal.pbio.2006738 30248089 PMC6171948

[22] Chen X. (2007). Challenges and solutions for stripe rust control in the united states. *Aust. J. Agric. Res*, 58(6):648–655. doi: 10.1071/AR07045

[23] Fukuoka S. and Okuno K. (2019). Strategies for breeding durable resistance to rice blast using pi21. *Crop Breeding*, *Genetics and Genomics*, 1(2).

[24] Flor H. H. (1971). Current status of the gene-for-gene concept. *Annu. Rev. Phytopathol*, 9(1):275–296. doi: 10.1146/annurev.py.09.090171.001423

[25] Vanderplank J. E. (1968). *Disease resistance in plants*. Academic Press.

[26] Sasaki A. (2000). Host-parasite coevolution in a multilocus gene-for-gene system. *Proc. R. Soc. B: Biol. Sci*, 267(1458):2183–2188. doi: 10.1098/rspb.2000.1267 PMC1690804 11413631

[27] Gandon S., van Baalen M., and Jansen V. A. (2002). The evolution of parasite virulence, superinfection, and host resistance. *Am. Nat*, 159(6):658–669. doi: 10.1086/339993 18707388

[28] Tellier A. and Brown J. K. (2007b). Stability of genetic polymorphism in host–parasite interactions. *Proc. Royal Soc. B*, 274(1611):809–817. doi: 10.1098/rspb.2006.0281 17251091 PMC2093977

[29] Fabre F., Rousseau E., Mailleret L., and Moury B. (2012). Durable strategies to deploy plant resistance in agricultural landscapes. *New Phytol*, 193(4):1064–1075. doi: 10.1111/j.1469-8137.2011.04019.x 22260272

[30] Nilusmas S., Mercat M., Perrot T., Djian-Caporalino C., Castagnone-Sereno P., Touzeau S., and al (2020). Multi-seasonal modelling of plant-nematode interactions reveals efficient plant resistance deployment strategies. *Evol. Appl*, 13(9):2206–2221. doi: 10.1111/eva.12989 33005219 PMC7513734

[31] Hamelin F., Mammeri Y., Aigu Y., Strelkov S., and Lewis M. (2022). Host diversification may split epidemic spread into two successive fronts advancing at different speeds. *Bull. Math. Biol*, 84(7):1–24. doi: 10.1007/s11538-022-01023-5 35598221

[32] Cruz C. M. V., Bai J., Oña I., Leung H., Nelson R. J., Mew T.-W., and al (2000). Predicting durability of a disease resistance gene based on an assessment of the fitness loss and epidemiological consequences of avirulence gene mutation. *Proc. Natl. Acad. Sci*, 97(25):13500–13505. doi: 10.1073/pnas.250271997 11095723 PMC17604

[33] Wichmann G. and Bergelson J. (2004). Effector genes of *Xanthamonas axonopodis* pv. *vesicatoria* promote transmission and enhance other fitness traits in the field. *Genetics*, 166(2):693–706. doi: 10.1534/genetics.166.2.693 15020460 PMC1470734

[34] Carson M. (1998). Aggressiveness and perennation of isolates of *Cochliobolus heterostrophus* from North Carolina. *Plant Dis*, 82(9):1043–1047. doi: 10.1094/PDIS.1998.82.9.1043 30856833

[35] Thrall P. H. and Burdon J. J. (2003). Evolution of virulence in a plant host-pathogen metapopulation. *Science*, 299(5613):1735–1737. doi: 10.1126/science.1080070 12637745

[36] Bahri B., Kaltz O., Leconte M., de Vallavieille-Pope C., and Enjalbert J. (2009). Tracking costs of virulence in natural populations of the wheat pathogen, *Puccinia striiformis* f. sp. *tritici*. *BMC Evol. Biol*, 9(1):26. doi: 10.1186/1471-2148-9-26 19183485 PMC2660305

[37] Huang Y.-J., Balesdent M.-H., Li Z.-Q., Evans N., Rouxel T., and Fitt B. D. (2010). Fitness cost of virulence differs between the Avrlm1 and Avrlm4 loci in *Leptosphaeria maculans* (phoma stem canker of oilseed rape). *Eur. J. Plant Pathol*, 126(2):279. doi: 10.1007/s10658-009-9539-7

[38] Caffier V., Didelot F., Pumo B., Causeur D., Durel C., and Parisi L. (2010). Aggressiveness of eight *Venturia inaequalis* isolates virulent or avirulent to the major resistance gene Rvi6 on a non-Rvi6 apple cultivar. *Plant Pathol*, 59(6):1072–1080. doi: 10.1111/j.1365-3059.2010.02345.x

[39] Bruns E., Carson M. L., and May G. (2014). The jack of all trades is master of none: a pathogen’s ability to infect a greater number of host genotypes comes at a cost of delayed reproduction. *Evolution*, 68(9):2453–2466. doi: 10.1111/evo.12461 24890322

[40] Bousset L., Sprague S. J., Thrall P. H., and Barrett L. G. (2018). Spatio-temporal connectivity and host resistance influence evolutionary and epidemiological dynamics of the canola pathogen *Leptosphaeria maculans*. *Evol. Appl*, 11(8):1354–1370. doi: 10.1111/eva.12630 30151045 PMC6099830

[41] Jenner C. E., Wang X., Ponz F., and Walsh J. A. (2002). A fitness cost for Turnip mosaic virus to overcome host resistance. *Virus Res*, 86(1-2):1–6. doi: 10.1016/S0168-1702(02)00031-X 12076824

[42] Janzac B., Montarry J., Palloix A., Navaud O., and Moury B. (2010). A point mutation in the polymerase of Potato virus Y confers virulence toward the Pvr4 resistance of pepper and a high competitiveness cost in susceptible cultivar. *Mol. Plant Microbe Interact*, 23(6):823–830. doi: 10.1094/MPMI-23-6-0823 20459321

[43] Fraile A., Pagán I., Anastasio G., Sáez E., and García-Arenal F. (2010). Rapid genetic diversification and high fitness penalties associated with pathogenicity evolution in a plant virus. *Mol. Biol. Evol*, 28(4):1425–1437. doi: 10.1093/molbev/msq327 21131559

[44] Poulicard N., Pinel-Galzi A., Hébrard E., and Fargette D. (2010). Why Rice yellow mottle virus, a rapidly evolving RNA plant virus, is not efficient at breaking rymv1-2 resistance. *Mol. Plant Pathol*, 11(1):145–154. doi: 10.1111/j.1364-3703.2009.00582.x 20078783 PMC6640461

[45] Ishibashi K., Mawatari N., Miyashita S., Kishino H., Meshi T., and Ishikawa M. (2012). Coevolution and hierarchical interactions of Tomato mosaic virus and the resistance gene Tm-1. *PLoS Pathog*, 8(10):e1002975. doi: 10.1371/journal.ppat.1002975 23093939 PMC3475678

[46] Khatabi B., Wen R.-H., and Hajimorad M. (2013). Fitness penalty in susceptible host is associated with virulence of soybean mosaic virus on Rsv1-genotype soybean: a consequence of perturbation of HC-Pro and not P3. *Mol. Plant Pathol*, 14(9):885–897. doi: 10.1111/mpp.12054 23782556 PMC6638797

[47] Djian-Caporalino C., Palloix A., Fazari A., Marteu N., Barbary A., Abad P., and al (2014). Pyramiding, alternating or mixing: comparative performances of deployment strategies of nematode resistance genes to promote plant resistance efficiency and durability. *BMC Plant Biol*, 14(1):1–13. doi: 10.1186/1471-2229-14-53 24559060 PMC3944934

[48] Montarry J., Hamelin F. M., Glais I., Corbière R., and Andrivon D. (2010). Fitness costs associated with unnecessary virulence factors and life history traits: evolutionary insights from the potato late blight pathogen *Phytophthora infestans*. *BMC Evol. Biol*, 10(1):283. doi: 10.1186/1471-2148-10-283 20846405 PMC2949872

[49] Groth J. (1976). Multilines and “super races”: a simple model. *Phytopathology*, 66(9).

[50] Marshall D. and Pryor A. (1978). Multiline varieties and disease control. *Theor. Appl. Genet*, 51(4):177–184. doi: 10.1007/BF00273143 24317749

[51] Leonard K. and Czochor R. (1980). Theory of genetic interactions among populations of plants and their pathogens. *Annual Review of Phytopathology*, 18(1):237–258. doi: 10.1146/annurev.py.18.090180.001321

[52] Kiyosawa S. (1982). Genetics and epidemiological modeling of breakdown of plant disease resistance. *Annu. Rev. Phytopathol*, 20(1):93–117. doi: 10.1146/annurev.py.20.090182.000521

[53] Ostergaard H. (1983). Predicting development of epidemics on cultivar mixtures. *Phytopathology*, 73:166–172.. doi: 10.1094/Phyto-73-166

[54] Segarra J. (2005). Stable polymorphisms in a two-locus gene-for-gene system. *Phytopathology*, 95(7):728–736. doi: 10.1094/PHYTO-95-0728 18943003

[55] Tellier A. and Brown J. K. (2007a). Polymorphism in multilocus host–parasite coevolutionary interactions. *Genetics*, 177(3):1777–1790. doi: 10.1534/genetics.107.074393 17947440 PMC2147965

[56] Ross A. F. (1961). Systemic acquired resistance induced by localized virus infections in plants. *Virology*, 14(3):340–358. doi: 10.1016/0042-6822(61)90318-X 13743578

[57] Maleck K., Levine A., Eulgem T., Morgan A., Schmid J., Lawton K. A., and al (2000). The transcriptome of *Arabidopsis thaliana* during systemic acquired resistance. *Nat. Genet*, 26(4):403. doi: 10.1038/82521 11101835

[58] Kuć J. (1982). Induced immunity to plant disease. *Bioscience*, 32(11):854–860. doi: 10.2307/1309008

[59] Fu Z. Q. and Dong X. (2013). Systemic acquired resistance: turning local infection into global defense. *Annu. Rev. Plant Biol*, 64:839–863.. doi: 10.1146/annurev-arplant-042811-105606 23373699

[60] Neuenschwander U., Lawton K., and Ryals J. (1997). Systemic acquired resistance. *Plant-microbe interactions*, pages 81–106. doi: 10.1007/978-1-4613-1213-0_3

[61] Madden L. V., Hughes G., and Van Den Bosch F. (2007). *The study of plant disease epidemics*. American Phytopathology Society.

[62] Armstrong R. A. and McGehee R. (1980). Competitive exclusion. *The American Naturalist*, 115(2):151–170. doi: 10.1086/283553

[63] Wuest S. E., Peter R., and Niklaus P. A. (2021). Ecological and evolutionary approaches to improving crop variety mixtures. *Nat. Ecol. Evol*, 5(8):1068–1077. doi: 10.1038/s41559-021-01497-x 34211140

[64] Rimbaud L., Fabre F., Papaïx J., Moury B., Lannou C., Barrett L. G., and al (2021). Models of plant resistance deployment. *Annu. Rev. Phytopathol*, 59:125–152. doi: 10.1146/annurev-phyto-020620-122134 33929880

[65] Ostfeld R. S. and Keesing F. (2012). Effects of host diversity on infectious disease. *Annu. Rev. Ecol. Evol. Syst*, 43(157):2012.

[66] Ben M’Barek S., Karisto P., Abdedayem W., Laribi M., Fakhfakh M., Kouki H., and al (2020). Improved control of septoria tritici blotch in durum wheat using cultivar mixtures. *Plant Pathol*, 69(9):1655–1665. doi: 10.1111/ppa.13247

[67] Ploetz R. C. (2007). Diseases of tropical perennial crops: challenging problems in diverse environments. *Plant Dis*, 91(6):644–663. doi: 10.1094/PDIS-91-6-0644 30780472

[68] Cox C., Garrett K., and Bockus W. (2005). Meeting the challenge of disease management in perennial grain cropping systems. *Renew. Agric. Food Syst*., 20(1):15–24. doi: 10.1079/RAF200495

[69] Sacristán S. and Garcia-arenal F. (2008). The evolution of virulence and pathogenicity in plant pathogen populations. *Mol. Plant. Pathol*, 9(3):369–384. doi: 10.1111/j.1364-3703.2007.00460.x 18705877 PMC6640236

