# Supplementary material for: The proportion of resistant hosts in mixtures should be biased towards the resistance with the lowest breaking cost
Source: PLoS Comput Biol. 2023 May 25;19(5):e1011146. doi: 10.1371/journal.pcbi.1011146 (PMC10246846; doi:10.1371/journal.pcbi.1011146)
Supplement: S1 Text — (PDF) [file pcbi.1011146.s001.pdf]

S1 Text: *Mathematical and numerical appendices to "The proportion of resistant hosts in mixtures should be biased towards the resistance with the lowest breaking cost".*

Pauline Clin, Frédéric Grognard, Didier Andrivon, Ludovic Mailleret, Frédéric M. Hamelin

Organisation of this document

This document consists of three sections:

- **S1. General model.** This section introduces the full model for host mixtures containing two resistant varieties. There are three possible pathogen genotypes: two monovirulent pathogens and a doubly virulent one. The model is therefore in dimension 6. While we can analyse to some extent this model, we next consider special a special case to get a better sense of its behaviour.
- **S2. Priming-less model ($\rho = 0$).** This special case can be more thoroughly analysed. In particular, we show that the system is cooperative in this case. This allows us to get a full understanding of the priming-less model.
- **S3. Bifurcation diagrams.** This section shows how the model behaves along the priming spectrum ($\rho \in [0,1]$). We first consider the priming-less ($\rho = 0$) and full-priming ($\rho = 1$) special cases. We then consider intermediate priming efficiencies ($\rho \in [0,1]$) in order to get a full picture of the model.
- **S4. Optimal mixture.** This section shows that one can get an explicit expression of the optimal proportion of resistance 2 in the mixture, $p^\star$, and how it depends on parameter values, in the biological reasonable case $c_1, c_2 < 0.5$.

S1 General model

First, we recall the meaning of the state variables in our model: y_i is the fraction of hosts of variety V_i infected by the monovirulent i pathogen genotype, for $i = 1,2$; z_i is the fraction of hosts of the resistant variety V_i infected by the doubly virulent pathogen genotype, for $i = 1,2$; m_i is the fraction of hosts of the resistant variety V_i primed by the monovirulent j pathogen genotype, for $i \neq j = 1,2$.

The dimensionless version of our model (Eq. 2 in the main text) is:

$$
\begin{aligned}
y_1' &= (1-c_1)Ry_1(1-p-m_1-y_1-z_1)+(1-\rho)(1-c_1)Ry_1m_1-y_1\,,\\
y_2' &= (1-c_2)Ry_2(p-m_2-y_2-z_2)+(1-\rho)(1-c_2)Ry_2m_2-y_2\,,\\
z_1' &= (1-c_1)(1-c_2)R(z_1+z_2)(1-p-m_1-y_1-z_1)+(1-\rho)(1-c_1)(1-c_2)R(z_1+z_2)m_1-z_1\,,\\
z_2' &= (1-c_1)(1-c_2)R(z_1+z_2)(p-m_2-y_2-z_2)+(1-\rho)(1-c_1)(1-c_2)R(z_1+z_2)m_2-z_2\,,\\
m_1' &= (1-c_2)Ry_2(1-p-m_1-y_1-z_1)-(1-\rho)(1-c_1)Ry_1m_1-(1-\rho)(1-c_1)(1-c_2)R(z_1+z_2)m_1-\nu m_1\,,\\
m_2' &= (1-c_1)Ry_1(p-m_2-y_2-z_2)-(1-\rho)(1-c_2)Ry_2m_2-(1-\rho)(1-c_1)(1-c_2)R(z_1+z_2)m_2-\nu m_2\,.
\end{aligned}
\tag{S1}
$$

The prevalence of the disease is defined as

$$P = y_1 + y_2 + z_1 + z_2\,.$$

For readibility in the model analysis, we let

$$R_1 = (1-c_1)R\,, \quad R_2 = (1-c_2)R\,, \text{ and } \quad R_3 = (1-c_1)(1-c_2)R\,.$$

S1.1 Equilibria

There are seven biologically feasible equilibria.

More specifically, there are 10 possible equilibria but three of them are biologically unfeasible. One equilibrium has the form $(0,0,z_1,z_2,m_1,m_2)$ and is biologically unfeasible because doubly virulent pathogens (z_1,z_2) do not prime hosts. The other two equilibria have the form $(y_1,0,0,0,m_1,m_2)$ and $(0,y_2,0,0,m_1,m_2)$. They are biologically unfeasible because y_1 cannot "prime" host V_1 to produce m_1 and y_2 cannot "prime" host V_2 to produce m_2. Therefore, these three equilibria must be out of the biologically relevant domain (*i.e.* $[0,1]^6$).

In particular, there exists no equilibrium of the form $(y_1,y_2,z_1,z_2,m_1,m_2) > 0$,

in which the three possible pathogen genotypes coexist. To show this, we notice that such an equilibrium would imply

$$\frac{y_1'}{R_1 y1} + \frac{y_2'}{R_2 y_2} - \frac{z_1' + z_2'}{R_3(z_1 + z_2)} = -\frac{1}{R_1} - \frac{1}{R_2} + \frac{1}{R_3} = 0\,,$$

which is not true in general.

S1.1.1 The "disease-free" equilibrium

The "disease-free" equilibrium $(0,0,0,0,0,0)$ always exists.

S1.1.2 The "monovirulent 1" equilibrium

The "monovirulent 1" equilibrium is $(y_1, 0, 0, 0, 0, m_2)$, with

$$y_1 = 1 - p - \frac{1}{R_1} \quad \text{and} \quad m_2 = \frac{p\,((1-p)R_1 - 1)}{(1-p)R_1 - 1 + \nu}\,,$$

where $y_1, m_2 > 0$ iff $(1-p)R_1 > 1$. The prevalence is:

$$P = y_1 = 1 - p - \frac{1}{R_1}\,. \tag{S2}$$

S1.1.3 The "monovirulent 2" equilibrium

The "monovirulent 2" equilibrium is $(0, y_2, 0, 0, m_1, 0)$, with

$$y_2 = p - \frac{1}{R_2} \quad \text{and} \quad m_1 = \frac{(1-p)(pR_2 - 1)}{pR_2 - 1 + \nu}\,,$$

where $y_2, m_1 > 0$ iff $pR_2 > 1$. The prevalence is

$$P = y_2 = p - \frac{1}{R_2}\,.$$

S1.1.4 The "doubly virulent" equilibrium

The "doubly virulent" equilibrium is $(0, 0, z_1, z_2, 0, 0)$ with

$$z_1 = (1-p)\left(1 - \frac{1}{R_3}\right) \quad \text{and} \quad z_2 = p\left(1 - \frac{1}{R_3}\right)$$

52 where z_1, $z_2 > 0$ iff $R_3 > 1$. The prevalence is

$$P = z_1 + z_2 = 1 - \frac{1}{R_3}\,. \tag{S3}$$

53 S1.1.5 The “monovirulent 1 and doubly virulent” equilibrium

The “monovirulent 1 and doubly virulent” equilibrium is $(y_1, 0, z_1, z_2, 0, m_2)$. At this equilibrium, we have

$$\begin{aligned} y_1' = 0 \quad &\Leftrightarrow \quad (R_1(1-p)-1)\,y_1 = R_1 y_1 (y_1 + z_1)\,, \\ z_1' + z_2' = 0 \quad &\Leftrightarrow \quad (R_3 - 1)(z_1 + z_2) = R_3(z_1+z_2)(y_1+z_1) + \rho R_3 (z_1+z_2) m_2\,. \end{aligned}$$

Since both r.h.s. are positive, this imposes

$$R_1(1-p) > 1 \text{ and } R_3 > 1\,.$$

From the whole system, the equilibrium is

$$y_1 = \frac{((1-\rho)(1-p)R_1 + \rho + \nu - 1)\,((1-p)R_1 - R_3)}{(R_1 - R_3)\,((1-\rho)(1-p)R_1 + \nu - 1)}\,,$$

$$z_1 = \frac{((1-\rho)pR_3 - 1)\,(1-p)R_1^2 + ((1+(\nu+\rho-2)p)\,R_3 - \nu + 1)\,R_1 + R_3(\nu - 1)}{R_1(R_1 - R_3)\,((1-\rho)(1-p)R_1 + \nu - 1)}\,,$$

$$z_2 = \frac{((1-\rho)pR_3 - 1)\,(1-p)R_1^2 + ((1+(\nu+\rho-2)p)\,R_3 - \nu + 1)\,R_1 + R_3(\nu - 1)}{R_3 R_1\,((1-\rho)(1-p)R_1 + \nu - 1)}\,,$$

$$m_2 = \frac{(1-p)R_1 - R_3}{R_3\,((1-\rho)(1-p)R_1 + \nu - 1)}\,,$$

54 where all denominators are positive since $R_3 < R_1$ and $\nu \geq 1$. Hence, $y_1, m_2 > 0$ iff

$$R_1(1-p) > R_3 > 1\,. \tag{S4}$$

55 Moreover, $z_1, z_2 > 0$ iff:

$$\rho < \frac{(R_1(1-p) + \nu - 1)(pR_3R_1 + R_3 - R_1)}{R_3 R_1 p (R_1(1-p) - 1)} =: \hat{\rho}_1\,. \tag{S5}$$

A necessary condition for (S5) to be achievable is that its r.h.s is positive. Its denominator is positive, as well as the first factor of the numerator. The remaining condition is then

$$pR_3R_1 + R_3 - R_1 > 0 \Leftrightarrow R_3 > \frac{R_1}{pR_1 + 1}.$$

which can be achieved with (S4) for some R_3 as $R_1(1-p) > R_1/(pR_1+1)$ when $R_1(1-p) > 1$.

To sum up, the “monovirulent 1 and doubly virulent” equilibrium is biologically feasible ($y_1, z_1, z_2, m_2 > 0$) if and only if conditions (S4) and (S5) are satisfied.

The associated prevalence is

$$P = y_1 + z_1 + z_2 = \frac{(\nu+\rho-1)R_3 - (1-(1-\rho)R_3)(1-p)R_1 - \nu + 1}{R_3((1-\rho)(1-p)R_1 + \nu - 1)}. \tag{S6}$$

Note that in the special case $\rho = 0$ (priming-less model), $P = 1 - 1/R_3$, meaning that the prevalence is the same as in the doubly virulent equilibrium (S3).

S1.1.6 The “monovirulent 2 and doubly virulent” equilibrium

The “monovirulent 2 and doubly virulent” equilibrium is $(0, y_2, z_1, z_2, m_1, 0)$, that is the same as the “monovirulent 1 and doubly virulent” equilibrium, up to the permutation of subscripts 1 and 2, plus the substitution of p with $1-p$ (since $p_1 = 1-p$ and $p_2 = p$).

Let

$$\hat{\rho}_2 := \frac{(R_2p + \nu - 1)((1-p)R_3R_2 + R_3 - R_2)}{R_3R_2(1-p)(R_2p-1)}. \tag{S7}$$

This way, this equilibrium ($y_2, z_1, z_2, m_1 > 0$) is biologically feasible if and only if

$$R_2p > R_3 > 1,$$

and

$$\rho < \hat{\rho}_2.$$

The prevalence is

$$P = y_2 + z_1 + z_2 = \frac{(\nu+\rho-1)R_3 - (1-(1-\rho)R_3)pR_2 - \nu + 1}{R_3((1-\rho)pR_2 + \nu - 1)}. \tag{S8}$$

S1.1.7 The "monovirulent 1 and monovirulent 2" equilibrium

The "monovirulent 1 and monovirulent 2" equilibrium is $(y_1, y_2, 0, 0, m_1, m_2)$. We solved the equilibria of this form using a formal calculus software (Maple 2021.2). Letting $x_i = R_i m_i$, $i = 1, 2$, the equilibrium $(y_1, y_2, 0, 0, m_1, m_2)$ can be expressed as: for $i = 1, 2$,

$$\begin{aligned} m_i &= \frac{x_i}{R_i}, \\ y_1 &= 1 - p - \frac{1}{R_1} - \rho m_1, \\ y_2 &= p - \frac{1}{R_2} - \rho m_2. \end{aligned}$$

The values of x_i, $i = 1, 2$, can be respectively found by solving the quadratics $Q_1(x_1)$ and $Q_2(x_2)$, which are defined as: for $i = 1, 2$,

$$Q_i(x_i) = A x_i^2 + B_i x_i + C_i,$$

with

$$A = -(1-\rho)\rho\,\{(1-\rho)\,[(1-p)R_1 + pR_2] + 2(\nu - 1)\} < 0,$$

and

$$\begin{aligned} B_1 &= (1-\rho)^2(1-p)^2R_1^2 + (1-\rho)\,[(1-\rho)(pR_2 - 1) + 2(\nu-1)]\,(1-p)R_1 \\ &\quad + R_2 p(1-\rho)(2\rho + \nu - 1) + (\nu - 1)(\nu + 3\rho - 2), \\ C_1 &= R_1R_2(1-\rho)p^2 - \{[(1-\rho)R_1 + \nu + \rho - 1]\,R_2 + R_1\}\,p \\ &\quad + R_1 + \nu - 1. \end{aligned}$$

Symmetrically,

$$\begin{aligned} B_2 &= (1-\rho)^2p^2R_2^2 + (1-\rho)\,[(1-\rho)((1-p)R_1 - 1) + 2(\nu-1)]\,pR_2 \\ &\quad + R_1(1-p)(1-\rho)(2\rho + \nu - 1) + (\nu - 1)(\nu + 3\rho - 2), \\ C_2 &= R_2R_1(1-\rho)(1-p)^2 - \{[(1-\rho)R_2 + \nu + \rho - 1]\,R_1 + R_2\}\,(1-p) \\ &\quad + R_2 + \nu - 1. \end{aligned}$$

The prevalence is

$$P = 1 - \frac{1}{R_1} - \frac{1}{R_2} - \rho(m_1 + m_2)\,.$$

The conditions $m_i > 0$ are equivalent to $x_i > 0$, for $i = 1, 2$. However, we have no simple condition for the positiveness of the roots of the quadratics. Exploratory numerical computations performed with several parameter sets suggest that only one of the two roots gives a result included in the biological space that makes sense, i.e. $y_1, m_1 \in (0, 1-p)$, and $y_2, m_2 \in (0, p)$.

Assuming $m_i > 0$ for $i = 1, 2$, $y_1 > 0$ and $y_2 > 0$ require, respectively:

$$R_1(1-p) > 1\,, \quad \text{and} \quad R_2 p > 1\,. \tag{S9}$$

To sum up, in the general case ($\rho \in [0, 1]$), we have no sufficient condition for the positiveness of the "monovirulent 1 and monovirulent 2" equilibrium, but only the above necessary conditions. However, we can get necessary and sufficient positiveness conditions in the special cases $\rho = 0$ and $\rho = 1$, as shown below.

Special case $\rho = 0$. Note that in the special case $\rho = 0$ (priming-less model), $A = 0$ and the quadratics simplify as linear equations of the form $B_i x_i + C_i = 0$, $i = 1, 2$, yielding:

$$\begin{aligned} x_1 &= -C_1/B_1 = \frac{R_2 p - 1}{(R_1(1-p)-1) + (R_2 p - 1) + \nu} > 0\,, \\ x_2 &= -C_2/B_2 = \frac{(1-p)R_1 - 1}{(R_1(1-p)-1) + (R_2 p - 1) + \nu} > 0\,. \end{aligned}$$

Therefore, $R_1(1-p) > 1$ and $R_2 p > 1$ are not only necessary but also sufficient conditions in this special case ($\rho = 0$), as they also ensure $y_i > 0$ and, since $x_i < 1$, $m_1 = \frac{x_1}{R_1} < 1 - p$ and $m_2 = \frac{x_2}{R_2} < p$.

Special case $\rho = 1$. Note that in the special case $\rho = 1$ (full-priming model), the following inequalities must be satisfied for the "monovirulent 1 and monovirulent

2” equilibrium to exist:

$$x_1 = -C_1/B_1 = \frac{\nu(R_2 p - 1) - [R_1(1-p) - 1]}{\nu^2 - 1} > 0\,,$$
$$x_2 = -C_2/B_2 = \frac{\nu[R_1(1-p) - 1] - (R_2 p - 1)}{\nu^2 - 1} > 0\,.$$

Since we assumed $R_1(1-p)-1 > 0$ and $R_2p-1 > 0$ (S9) the above pair of inequalities is equivalent to

$$\begin{aligned} \nu &> \frac{R_1(1-p)-1}{R_2p-1}\,, \\ \nu &> \frac{R_2p-1}{R_1(1-p)-1}\,. \end{aligned} \tag{S10}$$

One must also have $x_1 < R_1(1-p)$ (for $m_1 < 1-p$), $x_2 < R_2p$ (for $m_2 < p$) and $x_1 < R_1(1-p)-1$ (for $y_1 > 0$) and $x_2 < R_2p-1$ (for $y_2 > 0$). The latter two conditions are stronger than the former two, and are equivalent to (S10). Therefore, when ν is large enough, there exists a “monovirulent 1 and monovirulent 2” equilibrium.

S1.2 Local stability of general model equilibria

The Jacobian matrix can be expressed as:

$$J = \begin{pmatrix} A & 0 & B & 0 & C & 0 \\ 0 & D & 0 & E & 0 & F \\ G & 0 & H & I & J & 0 \\ 0 & K & L & M & 0 & N \\ O & P & Q & R & S & 0 \\ T & U & V & W & 0 & X \end{pmatrix}, \tag{S11}$$

in which the following notations are temporarily used:

$$
\begin{aligned}
A &= -1+(-m_1\rho-p-2y_1-z_1+1)R_1\,,\\
B &= -R_1y_1\,,\\
C &= -R_1y_1\rho\,,\\
D &= 1+(-m_2\rho+p-2y_2-z_2)R_2\,,\\
E &= -R_2y_2\,,\\
F &= -R_2y_2\rho\,,\\
G &= -R_3(z_1+z_2)\,,\\
H &= -1+(-m_1\rho-p-y_1-2z_1-z_2+1)R_3\,,\\
I &= -R_3(m_1\rho+p+y_1+z_1-1)\,,\\
J &= -R_3\rho(z_1+z_2)\,,\\
K &= -R_3(z_1+z_2)\,,\\
L &= R_3(-m_2\rho+p-y_2-z_2)\,,\\
M &= -1+(-m_2\rho+p-y_2-z_1-2z_2)R_3\,,\\
N &= -R_3\rho(z_1+z_2)\,,\\
O &= m_1(\rho-1)R_1-R_2y_2\\
P &= -R_2(-1+p+m_1+y_1+z_1)\,,\\
Q &= m_1(\rho-1)R_3-R_2y_2\\
R &= m_1(\rho-1)R_3\,,\\
S &= (\rho-1)(z_1+z_2)R_3+R_1y_1\rho-R_1y_1-R_2y_2-\nu\,,\\
T &= R_1(p-m_2-y_2-z_2)\,,\\
U &= m_2(\rho-1)R_2-R_1y_1\\
V &= (\rho-1)R_3m_2\,,\\
W &= (\rho-1)R_3m_2-R_1y_1\,,\\
X &= (\rho-1)(z_1+z_2)R_3+R_2y_2\rho-R_1y\,.
\end{aligned}
$$

S1.2.1 Disease-free equilibrium

The Jacobian matrix evaluated around the disease-free equilibrium $(0,0,0,0,0,0)$ is

$$J = \begin{pmatrix} R_1(1-p)-1 & 0 & 0 & 0 & 0 & 0 \\ 0 & R_2p-1 & 0 & 0 & 0 & 0 \\ 0 & 0 & R_3(1-p)-1 & R_3(1-p) & 0 & 0 \\ 0 & 0 & R_3p & R_3p-1 & 0 & 0 \\ 0 & R_2(1-p) & 0 & 0 & -\nu & 0 \\ R_1p & 0 & 0 & 0 & 0 & -\nu \end{pmatrix}. \qquad \text{(S12)}$$

Its eigenvalues are :

$$\begin{aligned} \lambda_1 &= -1 < 0\,, \\ \lambda_2 &= R_3 - 1\,, \\ \lambda_3 &= R_2p - 1\,, \\ \lambda_4 &= R_1(1-p) - 1\,, \\ \lambda_5 &= -\nu < 0\,, \\ \lambda_6 &= -\nu < 0\,. \end{aligned}$$

Therefore, the disease-free equilibrium is locally asymptotically stable if and only if $R_1(1-p) < 1$, $R_2p < 1$, and $R_3 < 1$. This means that the disease-free equilibrium is stable if and only if the basic reproductive numbers of the monovirulent 1, monovirulent 2, and doubly virulent genotypes are lower than 1.

S1.2.2 Monovirulent 1 equilibrium $(y_1, 0, 0, 0, 0, m_2)$

The eigenvalues of the Jacobian matrix (S11) evaluated around the "monovirulent 1" equilibrium $(y_1, 0, 0, 0, 0, m_2)$ are:

$$
\begin{aligned}
\lambda_1 &= -1 < 0\,,\\
\lambda_2 &= 1 - R_1(1-p)\,,\\
\lambda_3 &= 1 - R_1(1-p) - \nu < 0\,,\\
\lambda_4 &= 1 - R_1(1-\rho)(1-p) - \nu - \rho < 0\,,\\
\lambda_5 &= \frac{(1-p)(1-R_3(1-\rho)p)R_1^2 + ((-1+(2-\nu-\rho)p)R_3 + \nu - 1)R_1 - R_3(\nu-1)}{R_1(1-R_1(1-p)-\nu)}\,,\\
\lambda_6 &= \frac{R_1R_2(1-\rho)p^2 + ((-1-R_2(1-\rho))R_1 - R_2(\nu+\rho-1))p + R_1 + \nu - 1}{1-R_1(1-p)-\nu}\,.
\end{aligned}
$$

The $\lambda_2 < 0$ stability condition is simply the positivity condition of this equilibrium, i.e. $R_1(1-p) > 1$. With that, the other stability conditions, $\lambda_5 < 0$ and $\lambda_6 < 0$, are equivalent to, respectively:

$$\rho > \frac{(R_1(1-p)+\nu-1)(pR_3R_1 + R_3 - R_1)}{pR_3R_1(R_1(1-p)-1)} = \hat{\rho}_1\,,$$

and

$$\rho > \frac{(R_1(1-p)+\nu-1)(R_2p-1)}{R_2p(R_1(1-p)-1)} =: \check{\rho}_1\,. \tag{S13}$$

Therefore, the "monovirulent 1" equilibrium is stable if and only if

$$\rho > \max(\hat{\rho}_1, \check{\rho}_1)\,.$$

We have

$$\hat{\rho}_1 - \check{\rho}_1 = \frac{(R_1(1-p)+\nu-1)(R_2R_3 - R_1(R_2-R_3))}{pR_3R_2R_1(R_1(1-p)-1)}$$

Therefore,

$$\hat{\rho}_1 - \check{\rho}_1 > 0 \Leftrightarrow R_2R_3 - R_1(R_2-R_3) > 0 \Leftrightarrow c_2 + c_1 < 1\,.$$

This means that whether $\max(\hat{\rho}_1, \check{\rho}_1)$ equals $\hat{\rho}_1$ or $\check{\rho}_1$ only depends on the values of c_1 and c_2.

Note that the condition $\rho > \hat{\rho}_1$ is the opposite of inequality (S5), meaning that

a necessary condition for the “monovirulent 1” equilibrium to be stable is that the “monovirulent 1 and doubly virulent” equilibrium does not exist.

Exploratory numerical computations performed with several parameter sets suggest that $\rho > \check{\rho}_1$ prevents the existence of the “monovirulent 1 and monovirulent 2” equilibrium. In other words, the existence of either the “monovirulent 1 and doubly virulent” equilibrium or the “monovirulent 1 and monovirulent 2” would mean the “monovirulent 1” equilibrium is unstable.

S1.2.3 Monovirulent 2 equilibrium

The stability conditions of the “monovirulent 2” equilibrium $(0, y_2, 0, 0, m_1, 0)$ are the same as those of the “monovirulent 1” equilibrium $(y_1, 0, 0, 0, 0, m_2)$, up to the permutation of subscripts 1 and 2, plus the substitution of p with $1-p$ (since $p_1 = 1-p$ and $p_2 = p$).

Therefore, the “monovirulent 2” equilibrium is stable if and only if

$$\rho > \max(\hat{\rho}_2, \check{\rho}_2)\,,$$

with $\hat{\rho}_2$ as defined in equation (S7), and

$$\check{\rho}_2 := \frac{(R_2 p + \nu - 1)(R_1(1-p)-1)}{R_1(1-p)(R_2 p - 1)}\,. \tag{S14}$$

S1.2.4 Doubly virulent equilibrium

The Jacobian matrix evaluated around the “doubly virulent” equilibrium, that is $(0, 0, z_1, z_2, 0, 0)$, is:

$$J = \begin{pmatrix} \frac{R_1(1-p)-R_3}{R_3} & 0 & 0 & 0 & 0 & 0 \\ 0 & \frac{R_2 p - R_3}{R_3} & 0 & 0 & 0 & 0 \\ 1-R_3 & 0 & 1-p-R_3 & 1-p & -(R_3-1)\rho & 0 \\ 0 & 1-R_3 & p & p-R_3 & 0 & -(R_3-1)p \\ 0 & \frac{R_2(1-p)}{R_3} & 0 & 0 & (R_3-1)\rho - R_3 - \nu + 1 & 0 \\ \frac{R_1 p}{R_3} & 0 & 0 & 0 & 0 & (R_3-1)\rho - R_3 - \nu + 1 \end{pmatrix}. \tag{S15}$$

Its eigenvalues are :

$$\begin{aligned}
\lambda_1 &= -R_3 < 0\,,\\
\lambda_2 &= 1-R_3\,,\\
\lambda_3 &= \frac{R_2 p - R_3}{R_3}\,,\\
\lambda_4 &= \frac{R_1(1-p)-R_3}{R_3}\,,\\
\lambda_5 &= 1-R_3(1-\rho)-\nu-\rho < 0\,,\\
\lambda_6 &= 1-R_3(1-\rho)-\nu-\rho < 0\,.
\end{aligned}$$

The $\lambda_2 < 0$ stability condition is simply the positivity condition of this equilibrium, i.e. $R_3 > 1$. Therefore, the doubly virulent equilibrium is locally asymptotically stable if and only if

$$R_3 > R_2 p\,, \quad R_3 > R_1(1-p)\,.$$

This means that for the doubly virulent equilibrium to be stable, the basic reproductive number of the doubly virulent genotype has to be greater than the basic reproductive numbers of the monovirulent genotypes.

The last two conditions imply the nonexistence of “monovirulent and doubly virulent” equilibria, and are equivalent to $c_2 < p$ and $c_1 < 1-p$, respectively. This can be equivalently expressed as $c_2 < p < 1-c_1$. Therefore,

$$c_1 + c_2 < 1 \qquad \text{(S16)}$$

is a necessary condition for the doubly virulent equilibrium to be stable.

S1.3 Synthesis

Table A summarizes the equilibria, their positiveness, and their stability.

We were not able to derive explicit stability conditions for the “monovirulent i and doubly virulent” equilibria ($i = 1, 2$), and the “monovirulent 1 and monovirulent 2” equilibrium.

In the following section, we explore whether we can get explicit stability condi-

#	Equilibrium	Positiveness conditions	Extra Stability conditions
1	$(0,0,0,0,0,0)$	None	$R_1(1-p)\,,\, R_2p\,,\, R_3 < 1$
2	$(y_1,0,0,0,0,m_2)$	$R_1(1-p) > 1$	$\rho > \hat{\rho}_1\,,\, \check{\rho}_1$
3	$(0,y_2,0,0,m_1,0)$	$R_2p > 1$	$\rho > \hat{\rho}_2\,,\, \check{\rho}_2$
4	$(0,0,z_1,z_2,0,0)$	$R_3 > 1$	$R_3 > R_1(1-p)\,,\, R_2p$
5	$(y_1,0,z_1,z_2,0,m_2)$	$R_1(1-p) > R_3 > 1$ and $\rho < \hat{\rho}_1$	?
6	$(0,y_2,z_1,z_2,m_1,0)$	$R_2p > R_3 > 1$ and $\rho < \hat{\rho}_2$	?
7	$(y_1,y_2,0,0,m_1,m_2)$	$R_1(1-p)\,,\, R_2p > 1$ (!)	?

Table A: Summary of the equilibria of the general model (S1) and their stability. All conditions are necessary and sufficient conditions except those marked with an exclamation mark, which are only sufficient conditions in general. The expressions of $\hat{\rho}_1, \check{\rho}_1, \hat{\rho}_2, \check{\rho}_2$ are given by (S5) and (S7), respectively. The exclamation mark means that we have no explicit conditions in the general model.

tions for these equilibria in the special case $\rho = 0$ (priming-less model).

S2 Priming-less model

In this section, we focus on the priming-less special case, in which $\rho = 0$. Model S1 simplifies as

$$\begin{aligned} y_1' &= R_1 y_1(1-p-y_1-z_1) - y_1\,, \\ y_2' &= R_2 y_2(p-y_2-z_2) - y_2\,, \\ z_1' &= R_3(z_1+z_2)(1-p-y_1-z_1) - z_1\,, \\ z_2' &= R_3(z_1+z_2)(p-y_2-z_2) - z_2\,, \end{aligned} \tag{S17}$$

since m_1 and m_2 are not involved in these equations.

S2.1 The system is cooperative

The Jacobian matrix of the priming-less model is:

$$J = \begin{pmatrix} R_1(1-p-2y_1-z_1)-1 & 0 & -R_1y_1 & 0 \\ 0 & R_2(p-2y_2-z_2)-1 & 0 & -R_2y_2 \\ -R_3(z_1+z_2) & 0 & R_3(1-p-y_1-2z_1-z_2)-1 & R_3(1-p-y_1-z_1) \\ 0 & -R_3(z_1+z_2) & R_3(p-y_2-z_2) & R_3(p-y_2-z_1-2z_2)-1 \end{pmatrix}.$$

It has the following sign structure, where the asterisk means “any sign”:

$$J = \begin{pmatrix} * & 0 & - & 0 \\ 0 & * & 0 & - \\ - & 0 & * & + \\ 0 & - & + & * \end{pmatrix}$$

Since diagonal blocks have non-negative off-diagonal entries, and off-diagonal blocks have non-positive entries, the system is cooperative (1).

A cooperative system has the following feature: for almost all initial conditions, its solution converges to an equilibrium (2). As a corollary, there is no attractive periodic orbit.

S2.2 Stability of equilibria

In this section, we focus on the three equilibria for which we have no stability conditions in the general case (Table A). We have no explicit stability conditions for the “monovirulent i and doubly virulent” equilibria ($i = 1, 2$). Therefore, we focus on the “monovirulent 1 and monovirulent 2” equilibrium.

The Jacobian matrix evaluated around the “‘monovirulent 1 and monovirulent 2” equilibrium, i.e. $(y_1, y_2, 0, 0)$, is:

$$J = \begin{pmatrix} 1 - R_1(1-p) & 0 & 1-(1-p)R_1 & 0 \\ 0 & 1 - pR_2 & 0 & 1 - pR_2 \\ 0 & 0 & \frac{R_3}{R_1} - 1 & \frac{R_3}{R_1} \\ 0 & 0 & \frac{R_3}{R_2} & \frac{R_3}{R_2} - 1 \end{pmatrix}.$$

Its eigenvalues are:

$$\begin{aligned}
\lambda_1 &= -1 < 0\,, \\
\lambda_2 &= 1 - pR_2\,, \\
\lambda_3 &= 1 - R_1(1-p)\,, \\
\lambda_4 &= \frac{R_2R_3 + R_3R_1 - R_2R_1}{R_1R_2}\,.
\end{aligned}$$

The conditions of stability of the monovirulent pathogen genotypes equilibrium are

$$pR_2 > 1\,, \quad R_1(1-p) > 1\,, \quad \text{and} \quad R_3 < \frac{R_1R_2}{R_1+R_2}\,. \tag{S18}$$

The third condition is equivalent to

$$c_1 + c_2 > 1\,. \tag{S19}$$

S3 Bifurcation diagrams

As the mathematical analysis is incomplete (see Table A), this section is supported by an online interactive interface that numerically computes equilibria and their stability, and allows the user to test their own parameter sets: `https://share.streamlit.io/paulineclin/2_resistants_priming_model/main/app_chap3.py`.

S3.1 Priming-less model ($\rho = 0$)

Fig A shows bifurcation diagrams for the priming-less model ($\rho = 0$). We temporarily assume $c_1 = c_2$ for simplicity. Inequality S19 thus is equivalent to $c > 0.5$, meaning that the "monovirulent 1 and monovirulent 2" equilibrium is stable, and is otherwise ($c < 0.5$) unstable. Therefore, two cases are to be distinguished: $c > 0.5$ and $c < 0.5$.

- If $c < 0.5$, a two-dimensional bifurcation diagram in the plane (p, R) shows that all equilibria but the "monovirulent 1 and monovirulent 2" equilibrium can be stable, depending on parameter values. There is no multi-stability for

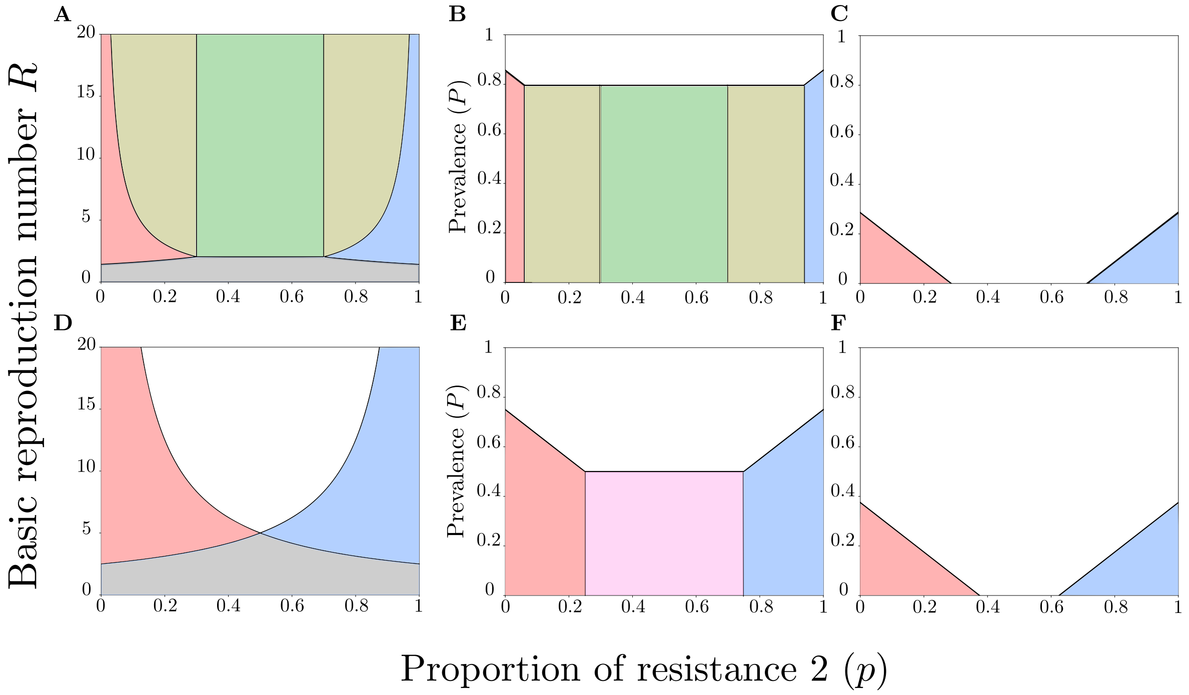

Fig A: Bifurcation diagrams from the priming-less ($\rho = 0$) model (S17), taking $c_1 = c_2$. The first row, in which $c = 0.3$, illustrates the case $c < 0.5$. In panels B and C, $R = 10$ and $R = 2$, respectively. The second row, in which $c = 0.6$, illustrates the case $c > 0.5$. In panels E and F, $R = 10$ and $R = 4$, respectively. The colored areas correspond to different genetic compositions of the pathogen population at equilibrium. The red color corresponds to the "monovirulent 1 only" equilibrium. The blue color corresponds to the "monovirulent 2 only" equilibrium. The yellow color corresponds to the "coexistence of monovirulent 1 or 2 and doubly virulent" equilibria. The green color corresponds to the "doubly virulent only" equilibrium. The pink color corresponds to the "coexistence of monovirulent 1 and 2" equilibrium. The grey color corresponds to the "disease free" equilibrium.

the parameter set considered, meaning that the stable equilibrium attracts all the trajectories (since the priming-less system is cooperative). In particular, the "monovirulent and doubly virulent" equilibria can be stable, meaning that coexistence of monovirulent and doubly virulent pathogens is possible in this model.

- Otherwise ($c > 0.5$), only four equilibria can be stable for the parameter set considered: the disease-free, the "monovirulent 1", the "monovirulent 2", and the "monovirulent 1 and monovirulent 2" equilibria. The coexistence of monovirulent and doubly virulent pathogens does not occur in this case.

In both cases ($c < 0.5$ and $c > 0.5$), the prevalence can be minimized by taking $p = 0.5$, meaning that the optimal ratio of disease-resistance plants is balanced.

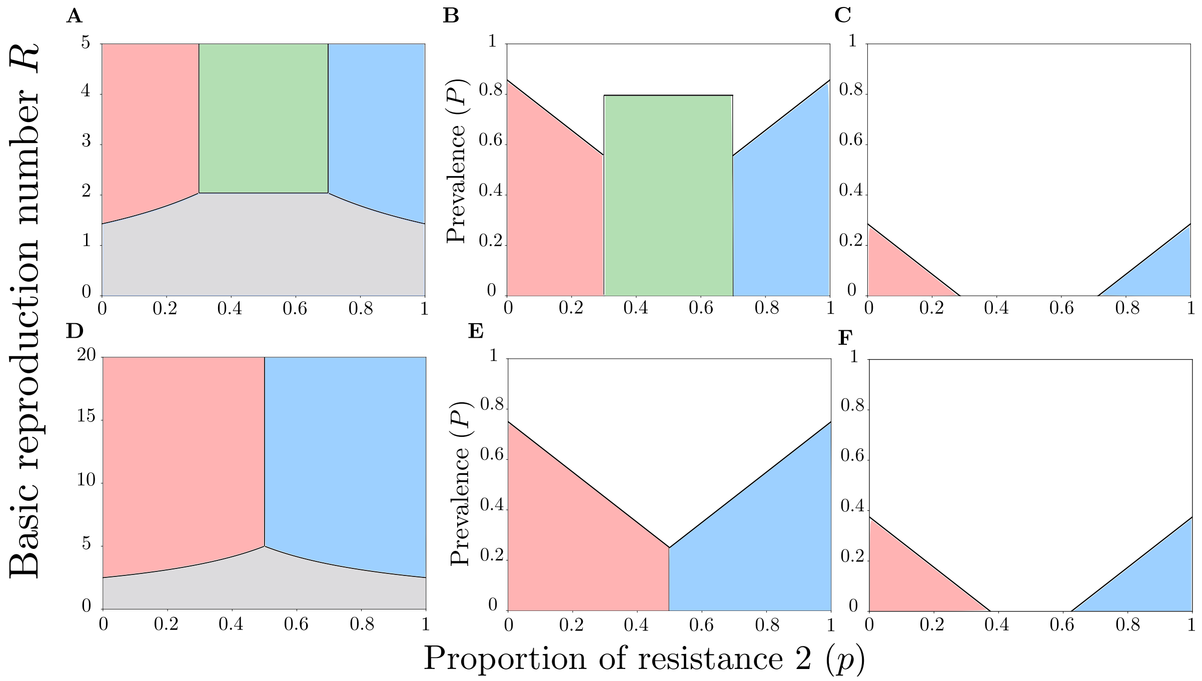

Fig B: Bifurcation diagrams from the full-priming ($\rho = 1$) model, taking $c_1 = c_2$, and $\nu = 1$. The first row, in which $c = 0.3$, illustrates the case $c < 0.5$. In panels B and C, $R = 10$ and $R = 2$, respectively. The second row, in which $c = 0.6$, illustrates the case $c > 0.5$. In panels E and F, $R = 10$ and $R = 2$, respectively. The colored areas correspond to different genetic compositions of the pathogen population at equilibrium, as detailed in Fig A's caption.

S3.2 Full-priming model ($\rho = 1$)

Fig B shows bifurcation diagrams analogous to Fig A ($\rho = 0$) for the full-priming ($\rho = 1$) model. We temporarily assume $c_1 = c_2$ for simplicity. Two cases are distinguished: $c > 0.5$ and $c < 0.5$.

- If $c < 0.5$, four equilibria can be stable, depending on parameter values: the disease-free, "monovirulent 1", "monovirulent 2", and "doubly virulent" equilibria. Monovirulent and doubly virulent pathogens can no longer coexist, as compared to Fig A ($\rho = 0$). Moreover, the prevalence can be minimized by taking $p \neq 0.5$ (i.e. $p = 0.5$ may be sub-optimal), meaning that the optimal ratio of disease-resistant plants may be imbalanced.

- If $c > 0.5$, three equilibria can be stable, depending on parameter values: the disease-free, "monovirulent 1", and "monovirulent 2" equilibria. The monovirulent pathogens can no longer coexist, as compared to Fig A ($\rho = 0$).

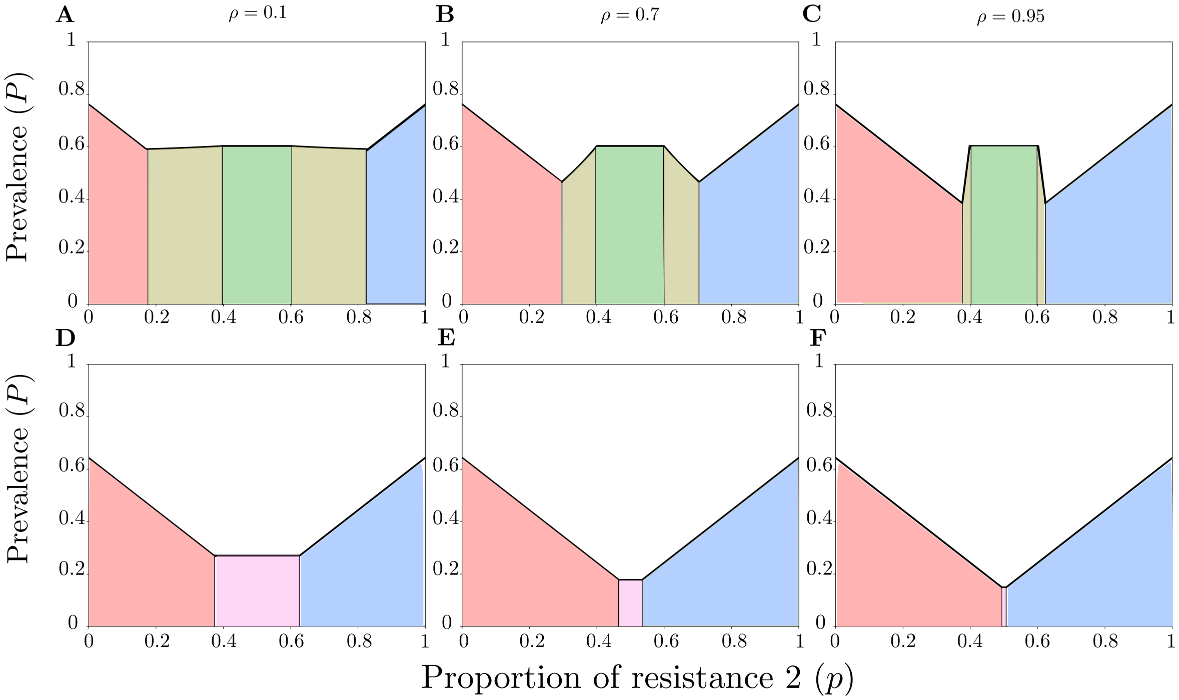

Fig C: Bifurcation diagrams for the general model (S1) with different values of the priming effect ($\rho = 0.1$, 0.7, and 0.95), taking $c_1 = c_2$. The first row, in which $c = 0.4$, illustrates the case $c < 0.5$. The second row, in which $c = 0.6$, illustrates the case $c > 0.5$. Other parameter values: $R = 7$ and $\nu = 1$. The colored areas correspond to different genetic compositions of the pathogen population at equilibrium, as detailed in Fig A's caption.

S3.3 Intermediate priming ($\rho \in (0,1)$)

S3.3.1 Symmetric case ($c_1 = c_2$)

Fig C shows bifurcation diagrams analogous to Figs A ($\rho = 0$) and B ($\rho = 1$) for intermediate priming ($\rho \in (0,1)$). The results illustrated in Fig C are representative of the results obtained with a broader range of parameter values, as shown in Fig D for $c_1 = c_2 > 0.5$, and Fig 3 of the main text for $c_1 = c_2 < 0.5$.

S3.3.2 Asymmetric case ($c_1 \neq c_2$)

Fig E shows bifurcation diagrams analogous to Fig C ($c_1 = c_2$) in the asymmetric case $c_1 \neq c_2$. This asymmetry leads to the existence of a unique optimal ratio of resistant plants that is imbalanced.

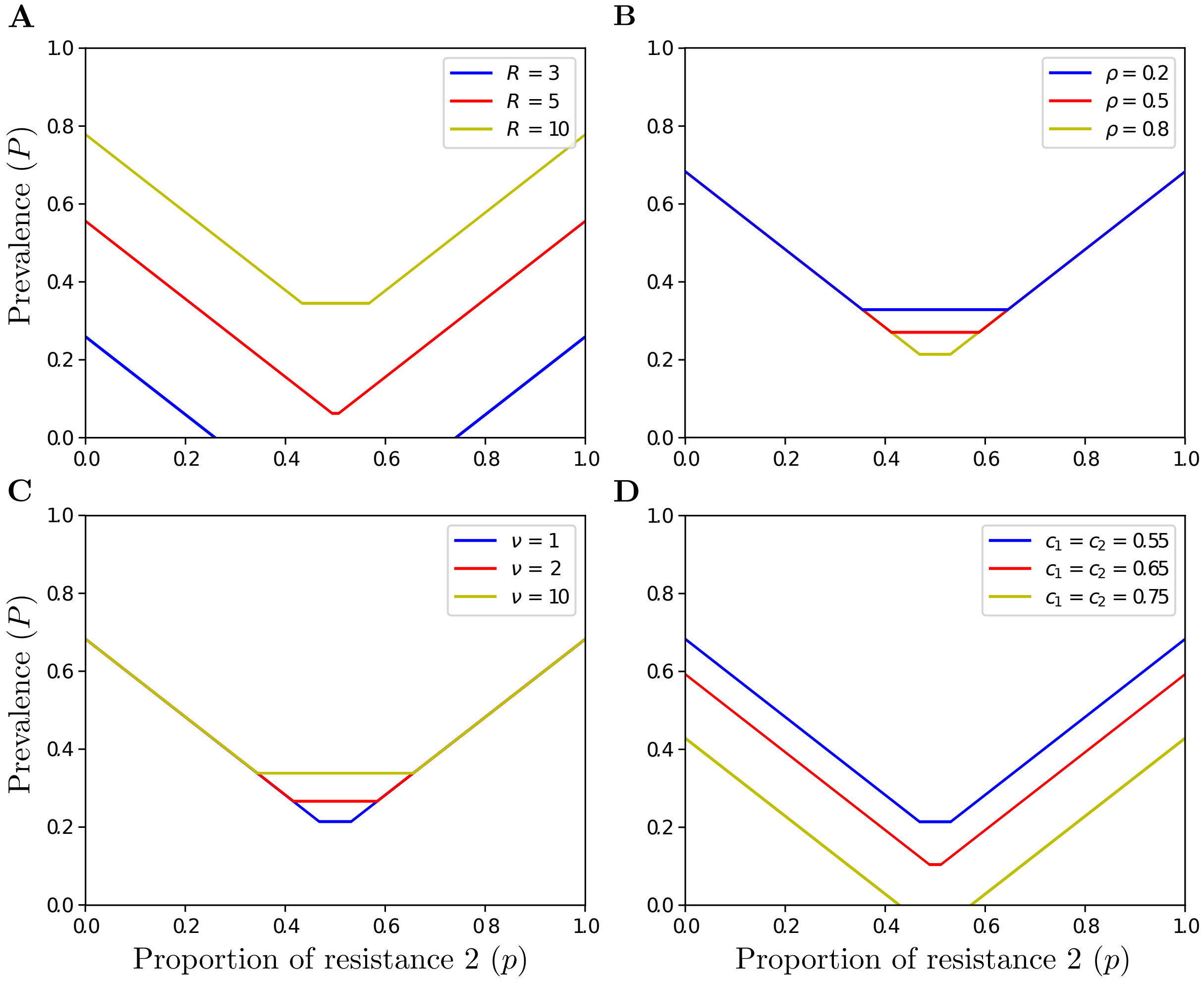

Fig D: Prevalence of the disease (P) as a function of the proportion of resistance 2 (p), when varieties are epidemiologically interchangeable ($c_1 = c_2 > 0.5$) for different values of (a) the basic reproductive number R, (b) the priming effect ρ, (c) the removal rate ν, and (d) the virulence costs c_1 and c_2. Parameter values: (a) $\rho = 0.8$, $\nu = 1$, and $c_1 = c_2 = 0.55$, (b) $R = 7$, $\nu = 1$, and $c_1 = c_2 = 0.55$, (c) $R = 7$, $\rho = 0.8$, and $c_1 = c_2 = 0.55$, (d) and $R = 7$, $\rho = 0.8$, and $\nu = 1$.

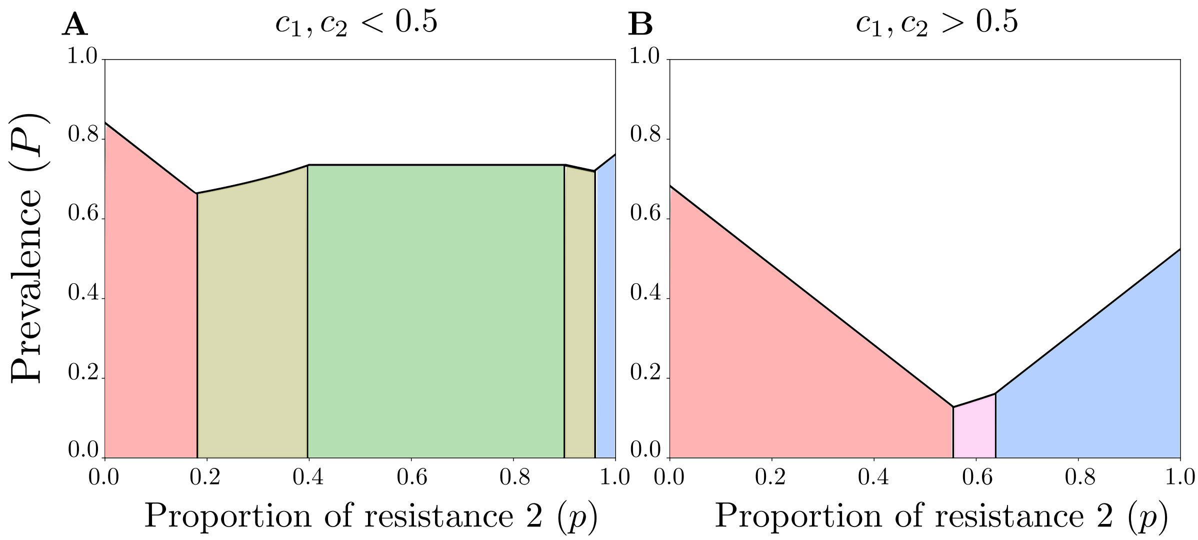

Fig E: Prevalence of the disease as a function of the proportion of resistant variety 2 in the mixture, for (a) $c_1 = 0.1$ and $c_2 = 0.4$, (b) $c_1 = 0.55$ and $c_2 = 0.7$. Other parameter values: $R = 7$, $\rho = 0.5$ and $\nu = 1$. The colored areas correspond to different genetic compositions of the pathogen population at equilibrium, as detailed in Fig A's caption.

S4 Optimal mixture

Let $p^\star$ be the optimal proportion of resistance 2. i.e. the proportion that minimizes prevalence.

When $c_1, c_2 < 0.5$, $p^\star$ is such that the prevalence associated with the "monovirulent 1" equilibrium (Eq. S2), equals the prevalence associated with the "monovirulent 1 and doubly virulent" equilibrium, (Eq. S6), see Fig EA. Equating equations (S2) and (S6) and solving for p amounts to solving the following quadratic equation:

$$Q(p) = Ap^2 + Bp + C = 0\,,$$

in which

$$\begin{aligned} A &= (1-\rho)R_3R_1 > 0\,, \\ B &= R_1 + (2 - 3(1-\rho)R_1 - \nu - \rho)R_3\,, \\ C &= ((-1 + 2(1-\rho)R_3)R_1^2 + (-R_3 - \nu + 1)R_1 - R_3(\nu - 1))/R_1\,. \end{aligned}$$

We notice that

$$Q(1) = A + B + C = (((-\nu - \rho + 1)R_3 - \nu + 1)R_1 - R_3(\nu - 1))/R_1 < 0\,,$$

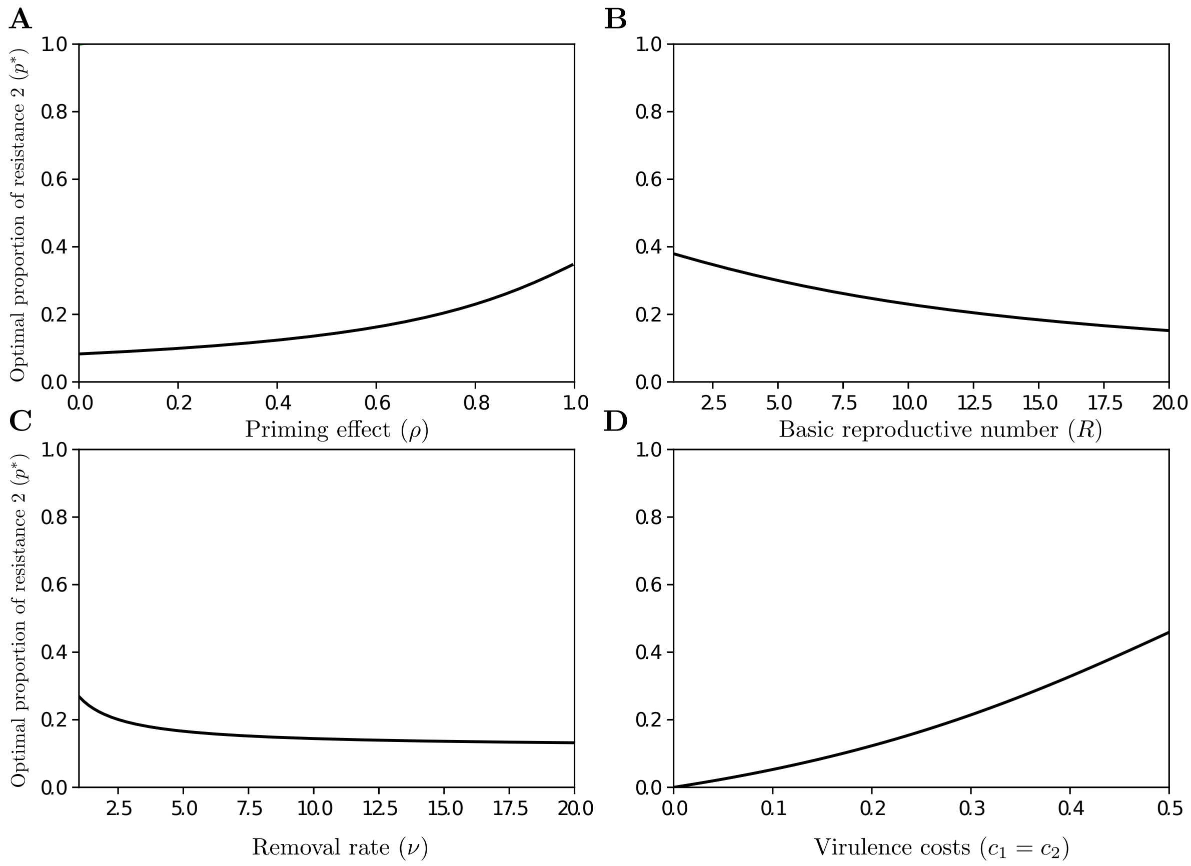

Fig F: The optimal proportion of resistance 2 ($p^\star$), when varieties are interchangeable ($c_1 = c_2$), as a function of (a) the priming effect (ρ), (b) the basic reproductive number (R), (c) the removal rate (ν), and (d) the virulence costs ($c_1 = c_2 < 0.5$). Parameter values: (a) $R = 10$, $c_1 = c_2 = 0.35$, and $\nu = 1$, (b) $\rho = 0.8$, $c_1 = c_2 = 0.35$, and $\nu = 1$, (c) $R = 7$, $\rho = 0.8$, and $c_1 = c_2 = 0.35$, (d) $R = 7$, $\rho = 0.8$, and $\nu = 1$.

since $\nu \geq 1$. Since $A > 0$, this means that there is necessarily one root greater than 1. The relevant root (such that $p^\star < 1$) is therefore the smallest one:

$$p^\star = \frac{-B - \sqrt{B^2 - 4AC}}{2A}.$$

We can hardly see how $p^\star$ varies with parameter values from this expression. Fig F shows how $p^\star$ depends on the parameters, for specific parameter sets.

When $c_1, c_2 > 0.5$, $p^\star$ is such that the prevalence associated with the "monovirulent 1" equilibrium equals the prevalence associated with the "monovirulent 1 and monovirulent 2" equilibrium (see Fig EB). Since we have no explicit expression of the latter prevalence (see Section S1.1.7), we can get no explicit expression of $p^\star$ in the $c_1, c_2 > 0.5$ case.

References

1. Smith HL. Monotone Dynamical Systems: An Introduction to the Theory of Competitive and Cooperative Systems: An Introduction to the Theory of Competitive and Cooperative Systems. 41. American Mathematical Soc.; 2008.

2. Hirsch MW. Systems of differential equations that are competitive or cooperative. V. Convergence in 3-dimensional systems. Journal of differential equations. 1989;80(1):94–106.
